# Deletion of Endocannabinoid Synthesizing Enzyme DAGLα in Pcp2-Positive Cerebellar Purkinje Cells Decreases Depolarization-Induced Short–Term Synaptic Plasticity, Reduces Social Preference, and Heightens Anxiety

**DOI:** 10.1523/ENEURO.0400-24.2025

**Published:** 2025-07-22

**Authors:** Gabriella Smith, Kathleen McCoy, Gonzalo Viana Di Prisco, Alexander Kuklish, Emma Grant, Mayil Bhat, Sachin Patel, Ken Mackie, Brady Atwood, Anna Kalinovsky

**Affiliations:** ^1^Gill Institute for Neuroscience, Indiana University, Bloomington, Indiana 47405; ^2^Program in Neuroscience, Department of Psychological and Brain Sciences, Indiana University, Bloomington, Indiana 47405; ^3^Department of Pharmacology and Toxicology, Indiana University School of Medicine, Indianapolis, Indiana 47405; ^4^Stark Neurosciences Research Institute, Indiana University School of Medicine, Indianapolis, Indiana 46402; ^5^Department of Molecular Physiology and Biophysics, Department of Psychiatry, Vanderbilt Brain Institute, Vanderbilt-Kennedy Center for Research on Human Development, Vanderbilt University School of Medicine, Nashville, Tennessee 37232; ^6^Center for Psychiatric Neuroscience, Department of Psychiatry and Behavioral Sciences, Northwestern University, Feinberg School of Medicine, Chicago, Illinois 60611; ^7^Department of Pharmacology, University of Minnesota Medical School, Minneapolis, Minnesota 55455

**Keywords:** cannabinoid, cerebellum, DAGLα, DSE, DSI, Purkinje

## Abstract

The endocannabinoid (eCB) signaling system is robustly expressed in the cerebellum from embryonic developmental stages to adulthood. It plays a key role in regulating cerebellar synaptic plasticity and excitability, suggesting that impaired eCB signaling could lead to deficits in cerebellar adjustments of ongoing behaviors and cerebellar learning. Indeed, human mutations in *DAGLα* are associated with neurodevelopmental disorders. In this study, we show that selective deletion of the eCB synthesizing enzyme diacylglycerol lipase alpha (Daglα) from mouse cerebellar Purkinje cells (PCs) alters motor and social behaviors, disrupts short-term synaptic plasticity in both excitatory and inhibitory synapses, and reduces PC activity during social exploration. Our results provide the first evidence for cerebellar-specific eCB regulation of social behaviors and implicate eCB regulation of synaptic plasticity and PC activity as the neural substrates contributing to these deficits.

## Graphical abstract

**Figure:**
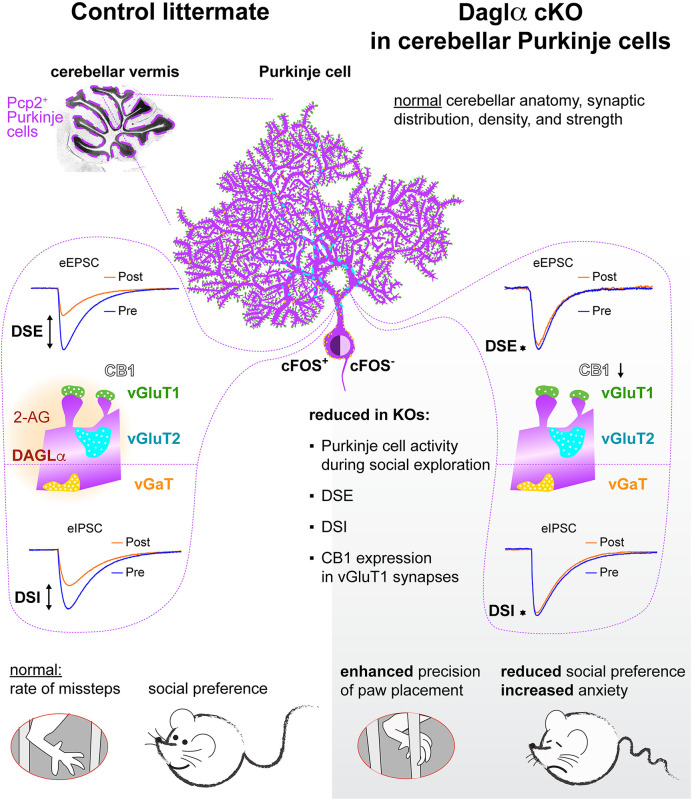
Visual Abstract

## Significance Statement

Deletion of the endocannabinoid synthesizing enzyme diacylglycerol lipase alpha from mouse cerebellar Purkinje cells (PCs) alters motor and social behaviors, disrupts short-term synaptic plasticity, and reduces PC activity during social exploration.

## Introduction

The cerebellum plays a key role in the regulation of implicit behaviors, ranging from motor coordination and balance to emotional processing (Buckner et al., 2011; Manto et al., 2012; Stoodley and Schmahmann, 2018; Van Overwalle et al., 2020). Cerebellar outputs are controlled Purkinje cell (PC) activity, which, in turn, is influenced by endocannabinoid (eCB) signaling (Maejima et al., 2001; Kreitzer and Regehr, 2001a; Kishimoto and Kano, 2006; Myoga and Regehr, 2013). eCB signaling machinery is prominently expressed in the cerebellum: diacylglycerol lipase alpha (Daglα), the primary synthesizing enzyme of 2-arachidonoylglycerol (2-AG), a major neuronal eCB, is highly expressed in cerebellar PCs (Bisogno et al., 2003; Tanimura et al., 2010), and cannabinoid receptor 1 (CB1), the main neuronal receptor for 2-AG, is expressed in the axons and the presynaptic terminals of both excitatory and inhibitory synaptic inputs to PCs (Kreitzer and Regehr, 2001b; Yoshida et al., 2002; Kawamura et al., 2006; Carey et al., 2011).

Synaptic plasticity—changes in synaptic strength that persist on a wide range of timescales, from milliseconds–minutes (short-term) to hours–days–years (long-term)—is the main substrate of learning and memory (Citri and Malenka, 2008). eCB signaling plays a key role in cerebellar long-term and short-term synaptic plasticity, referred to as long-term synaptic potentiation and depression (Safo and Regehr, 2005; van Beugen et al., 2006) and depolarization-induced suppression of excitation and inhibition (DSE and DSI), respectively (Kreitzer and Regehr, 2001a,b; Yoshida et al., 2002). It is tempting to suggest that eCB signaling could be a key mediator of cerebellar-dependent learning and behavior.

Studies on the neurodevelopmental roles of eCB signaling have focused on the fore- and midbrain, where developmental disruptions of eCB signaling result in axon misrouting, aberrant synaptic development, and motor, cognitive, and emotional deficits (Berghuis et al., 2005, 2007; Mulder et al., 2008; Wu et al., 2010; Guy et al., 2015; Khaliullina et al., 2015; Díaz-Alonso et al., 2017; Miller et al., 2019; Davies et al., 2022). The contribution of eCB signaling to cerebellar development is not well characterized, but we expected it to play a prominent role based on findings in other brain regions and on our recent work that showed robust expression of the eCB signaling system in the developing cerebellum. The hypothesis that eCB signaling contributes to the regulation of cerebellar development is further supported by the evidence of anatomical and behavioral abnormalities in global CB1 KOs—reduced size, altered morphology of anterior cerebellar vermis, and deficits in forelimb coordination (Martinez et al., 2020).

Mutations in *DAGLα* are associated with neurodevelopmental disorders and cerebellar ataxias in humans (Knight et al., 2008; Smith et al., 2017; Bainbridge et al., 2022) and are implicated in one of the most common neurodevelopmental diagnoses, autism spectrum disorder (ASD; Shonesy et al., 2014, 2018; Jenniches et al., 2016; Fyke et al., 2021). ASD diagnostic criteria (Carey, 2016) include reduced social interactions, increased repetitiveness, and atypical sensorimotor learning and performance—such as toe walking in toddlers, increased movement stereotypy, and sometimes extraordinary performance of technical and cognitive skills (Hughes et al., 2018; Leyden et al., 2019). Since many ASD-associated behaviors suggest cerebellar involvement, could cerebellar eCB signaling due to mutations in *DAGLα* lead to social, emotional, and motor behavioral deregulation associated with ASD?

This notion is supported by recent human gene association studies. A 2017 publication identified 35 individuals with rare genetic variants in *DAGLα* (the majority of the mutations were identified in the C-terminal tail of *DAGLα*) diagnosed with ASD, developmental delays, movement disorders, ataxia, and seizure disorders (Smith et al., 2017). A recent report in 2022 described nine children with heterozygous de novo truncating mutations in the C-terminal tail of *DAGLα* (affecting phosphorylation sites, Homer interactions, DAGLα localization, and on-demand production of 2-AG) who exhibit delayed motor and verbal-communication development together with the typical cerebellar symptoms, such as ataxia and nystagmus (Bainbridge et al., 2022). Together, these studies suggest that a reduction in DAGLα-dependent 2-AG production results in cerebellar dysfunction and ASD-associated behavioral deregulation in humans.

Anatomical lesion association studies support cerebellar involvement in ASD pathology, showing that neonatal cerebellar injury dramatically increases the predisposition for ASD (Wang et al., 2014), and PC loss is a common finding in ASD patients postmortem (Bailey et al., 1998). These findings motivated the generation of PC-specific knock–out (KO) mouse models of widely expressed ASD-associated genes, such as FMR1, SHANK2, and TSC1/2. These models exhibit reduced sociability and other ASD-associated behavioral phenotypes (Koekkoek et al., 2005; Tsai et al., 2012; Reith et al., 2013; Peter et al., 2016), suggesting a critical role for the cerebellum in mediating pathology associated with their mutations. However, the role of cerebellar PC Daglα in the regulation of cerebellar activity and cerebellar-influenced social and motor behaviors has not been specifically investigated before. To address this question, we generated PC-specific Daglα conditional KO mice to explore the function of Daglα in cerebellar PC synaptic development and plasticity and in cerebellar-influenced behaviors.

Our results show alterations in motor adjustments during horizontal ladder locomotion, social approach, and anxiety in the KO mice, while forelimb coordination and repetitive behaviors are unaffected, implicating cerebellar eCB signaling in the regulation of a specific subset of behavioral domains. Confocal microscopy and 3D volumetric reconstruction were used to evaluate synaptic distribution in Daglα KO PCs, and electrophysiology in cerebellar slices was used to evaluate short-term synaptic plasticity. Daglα KO PCs exhibit reduced CB1 expression in vGluT1-positive presynaptic terminals, while the overall distribution and density of presynaptic terminals onto PC dendrites and somata are normal. Cannabinoid-dependent short–term synaptic plasticity in both excitatory and inhibitory synapses is dramatically reduced in Daglα KO PCs. The overall activity of Daglα KO PCs during social exploration, as assessed by expression of the immediate early gene c-Fos, is reduced concomitantly to the reduced preference for exploring social cues.

## Materials and Methods

### Mouse colony

**Figure 1.**
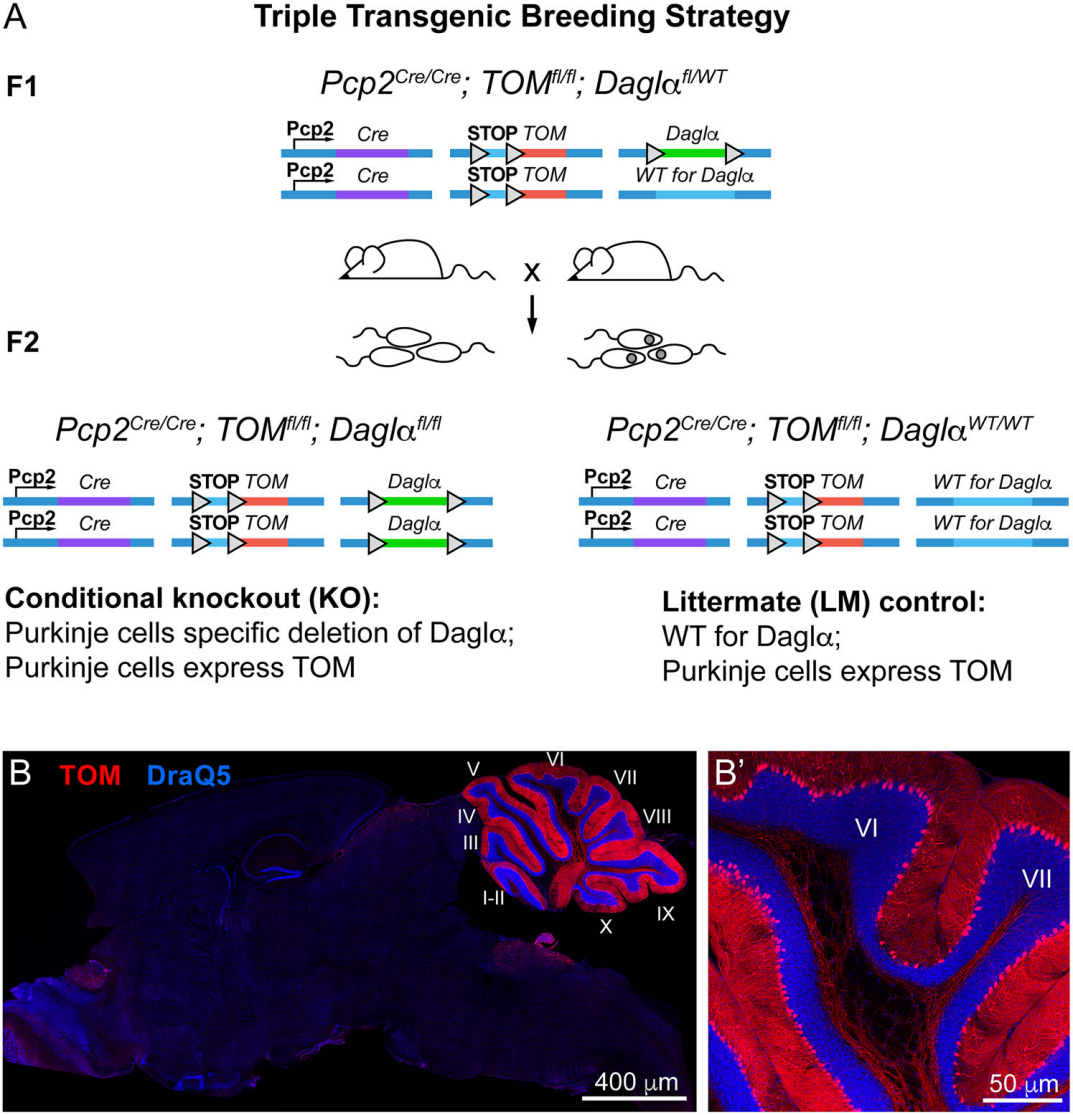
Pcp2^Cre^ recombination is selective to PCs. ***A***, LM controls, and conditional KO were generated by breeding Pcp2^Cre/Cre^; Ai9^fl/fl^; DAGLα^fl/WT^. The floxed TOM allele (Ai9 reporter) was included, allowing the visualization of recombined PCs with TOM expression. Triangles represent LoxP sites. F1, first generation, breeding; F2, offspring, the genotypes used in experiments. ***B***, ***B*’**, A representative midsagittal section from a 2-month-old mouse shows that reporter expression is highly restricted to the cerebellar PCs. ***B*’**, higher magnification of cerebellar lobules VI and VII.

All mice used in this study were maintained on an outbred CD1 genetic background. The CD1 IGS stock was acquired from Charles River Laboratories (https://www.criver.com/products-services/find-model/cd-1r-igs-mouse?region=3611) and replenished by purchasing additional CD1 breeders from Charles River Laboratories once a year. A breeding colony was maintained in the vivarium with a 12 h light/dark cycle under conditions stipulated by the Institutional Animal Care and Use Committee. The *Daglα* floxed (*Daglα^fl^*) mouse line was generated by the group of Dr. Sachin Patel (Bluett et al., 2017). The PC-specific Cre line (*Pcp2^Cre^*) was generated by the group of Noboru Suzuki (Saito et al., 2005). The Ai9 [B6.Cg-Gt(ROSA)26Sortm9(CAG-tdTomato)Hze/J] tdTomato (TOM) reporter mouse line was purchased from the Jackson Laboratory (https://www.jax.org/strain/007909). All mouse lines arrived in our colony on B6C57 genetic background and have been backcrossed to CD1 background in our colony for ≥7 generations. We generated triple transgenic mice by crossing the founder lines, as described in the results (Fig. 1*A*). First-degree crosses (siblings, parents, etc.) were always avoided. Both female and male mice were used in all experiments reported below. Sex-combined results are shown for the experiments where we observed no sex differences. However, the data points in the graphs are color-coded to show the distribution of results for both males and females.

### Tissue preparation

The brain tissue was fixed by transcardial perfusion with 1× PBS followed by 4% PFA. Dissected brains were postfixed in 4% PFA at 4°C overnight, switched to 0.2% sodium azide 1× PBS (PBS-Az), and stored at 4°C until sectioning. The brains were sectioned at 70 μm using a Leica VT 1000S vibrating microtome. Brains of newborn (P0) mice were immersion fixed in 4% PFA at 4°C overnight.

### Antibodies and immunohistochemistry

Immunohistochemistry was conducted on free-floating 70 μm tissue sections. The tissue sections were washed three times in 1× PBS and incubated in BSA blocking buffer (5% BSA, 1% Triton X-100, 1× PBS). Primary antibodies were applied overnight at 4°C in BSA blocking buffer. The tissue was washed three times in 1× PBS, and secondary antibodies (Alexa Flour 488, 596, or 647 from Jackson ImmunoResearch Laboratories) were applied at 4°C overnight (1:600 in BSA blocking buffer). DraQ5 (Cell Signaling Technology) was used (at 1:5,000 in 1× PBS) to visualize cell nuclei. Streptavidin Alexa Fluor 488 (Invitrogen) was used at 1:1,000 in BSA blocking buffer to visualize biocytin-filled neurons. Slides were coverslipped using Flouoromount G (SouthernBiotech). We used the primary antibodies found in Table 1.

**Table 1.** Primary antibodies

Antibody	Source	RRID	Working dilution
Guinea pig anti Daglα	Custom made, polyclonal (Katona et al., 2006)	AB_2813825	1:1,000
Rabbit anti Cre	Synaptic Systems, polyclonal, catalog #257003	AB_2619968	1:200
Mouse anti Plcβ4	Santa Cruz Biotechnology, monoclonal, clone A-8, catalog #sc-166131	AB_2237003	1:500
Rabbit anti vGluT1	Synaptic Systems, polyclonal, catalog #135302	AB_887877	1:2,000
Guinea pig anti-vGluT2	Synaptic Systems, polyclonal, catalog #135404	AB_887884	1:500
Rabbit anti-vGaT	Synaptic Systems, polyclonal, catalog #131013	AB_2189938	1:1,000
Guinea pig anti-CB1	Custom made, polyclonal (Berghuis, et al., 2007)	AB_2813824	1:250
Rabbit anti-cFos	Abcam, polyclonal, catalog #ab190289	AB_2737414	1:1,000

### Microscopy and image analysis

A Nikon A1 confocal microscope was used to collect confocal image *Z*-stacks. The cerebellar area and layer thickness were analyzed from DraQ5-stained cerebellar sagittal midvermal sections using the free-hand tool to measure areas of interest and distances in FiJi (Fiji). The FiJi cell counter tool was used to assess the numbers of cFos^+^ cells from max-intensity-projection 5-μm-thick confocal image stacks. For the analysis of synaptic density, a 60× objective was used to collect 5-μm-thick confocal image stacks from midvermal lobe VI. The density of synaptic puncta per surfaces of PC somata and dendrites was assessed in Imaris (Oxford Instruments). Regions of interest were manually outlined for PC somata (PC layer, PCL) and dendrites (molecular layer, ML). TOM expression in PCs was used to reconstruct the surfaces of somata and dendrites. Spots were generated based on vGluT1, vGluT2, and vGaT synaptic puncta staining and then filtered to show only those within 0.2 µm of the soma (yellow spots) and dendrite (turquoise spots) surfaces. These spots were classified as “on soma” or “on dendrites.” “Statistical analysis” was performed in GraphPad Prism 10 (www.graphpad.com). For weight and anatomy (midvermal area, ML width), breeding generation F3 mice, 9 littermate (LM) females, 15 LM males, 12 KO females, and 9 KO males were used (Fig. 3). Less than 10 mice per condition, chosen randomly, were perfused for microscopy analysis. Additional mice were excluded from microscopy analysis after quality control for sectioning and staining. The statistical hypothesis tested the magnitude of differences between KOs and LMs. An unpaired Student’s *T* test was used to evaluate means, SEMs, and *p* values. More details are shown in the statistics table (Table 2). Mice from breeding generations F3 and F4 (seven LM females, seven LM males, 10 KOs females, 6 KO males) were used to evaluate CB1 overlap with vGluT1 (Fig. 4). The same cohort of mice was used to assess vGluT1, vGluT2, and vGaT synaptic puncta density and distribution (Fig. 9). Not all mice were used in every experiment—details are provided in the statistics table (Table 2). In Figure 14, mice from breeding generations F3 and F4 (seven LM females, seven LM males, 10 KOs females, 6 KO males) were used to assess the density of cFos^+^ cells after the olfactory social choice test. Behavioral results (preference to explore “social cup,” locomotion in three-chamber arena and in open field) were collected for the same mice. The statistical hypotheses tested the magnitude of differences between KOs and LMs. Not all mice were used in every experiment—details are provided in the statistics table (Table 2). ANOVA or unpaired Student’s *T* tests, as appropriate, were used to evaluate means, SEMs, and *p* values. For the experiments most relevant to the hypothesis, estimation plots were used to analyze the CIs (CIs) for the differences between means.

**Table 2.** Statistics table

Figure number	Data structure	Type of test	*P* values, SEM, CIs	Number of biological replicates, means	Generation; have any data points been excluded and why?
Figure 3*F* (mouse weights at 2-month-old)	Normal distribution; males *F* test to compare variances, *F*, DFn, Dfd = 1.219, 8, 14; Females *F* test to compare variances, *F*, DFn, Dfd = 1.103, 11, 8	Unpaired *T* test	Females: *p* value of unpaired *T* test, 0.2644; the difference between means ± SEM = −0.6972 ± 0.6063; 95% CI = −1.966 to 0.5717; males: *p* value of unpaired *T* test, 0.8912; the difference between means ± SEM = 0.1467 ± 1.060; 95% CI = −2.052 to 2.345	Female: 9 LMs; mean, 25.02 g; 12 KOs; mean, 24.33 g; male: 15 LMs; mean, 31.92 g; 9 KOs mean, 32.07 g	Breeding generation F3
Figure 3*G* (midvermal area at 2-month-old)	Normal distribution; males: *F* test to compare variances, *F*, DFn, Dfd = 1.055, 6, 4; females: *F* test to compare variances, *F*, DFn, Dfd = 1.329, 8, 6	Unpaired *T* test	Females: *p* value of unpaired *T* test, 0.2208; the difference between means ± SEM = 0.5918 ± 0.4618, 95% CI = −0.3986 to 1.582; males: *p* value of unpaired *T* test, 0.1216; the difference between means ± SEM = 0.8899 ± 0.5260, 95% CI = −0.2822 to 2.062	Females: 7 LMs mean, 9.866 μm^2^, 9 KOs = 10.46 μm^2^; males: 7 LMs mean, 10.19 μm^2^, 5 KOs = 11.08 μm^2^	Breeding generation F3; same mice as in Figure 2*F*. Not more than 10 mice per condition have been perfused due to time constrains (i.e., 2 KO Fs and 5 LM Ms have been excluded randomly). Additional mice have been excluded from the analysis after sectioning and staining if no intact sections encompassing entire midvermis (without tears or folding) were available
Figure 3*H* (ML width at 2-month-old)	Males: normal distribution; *F* test to compare variances, *F*, DFn, Dfd = 1.165, 5, 6. females: *F* test to compare variances revealed that the distribution of values is significantly tighter in KOs than in LMs. The tight distribution of values in KO females is atypical as compared with the wider spread of values in all other conditions. Females *F* test to compare variances: *F*, DFn, Dfd = 6.020, 6, 9	Unpaired *T* test	Females: *p* value of unpaired *T* test, 0.1484; the difference between means ± SEM = 16.42 ± 10.78, 95% CI = −6.550 to 39.39; males: *p* value of unpaired *T* test, 0.1896; the difference between means ± SEM = 15.80 ± 11.30; 95% CI = −9.071 to 40.68	Females: 7 LMs; mean, 162.5 μm; 10 KOs = 178.9 μm; males: 7 LMs; mean, 169.0 μm; 6 KOs = 184.8 μm	Breeding generation F3; same mice as in Figure 2*F*. Not more than 10 mice per condition have been perfused due to time constrains (i.e., 2 KO Fs and 5 LM Ms have been excluded randomly). Additional mice have been excluded from the analysis after sectioning and staining if no intact sections of midvermal lobe VI (without tears or folding) were available
Figure 4*E* (ratio of CB1 over ML ROI)	Normal distribution. *F* test to compare variances, *F*, DFn, Dfd = 3.473, 8, 11	Unpaired *T* test	*P* value of unpaired *T* test, 0.0231; the difference between means ± SEM = −0.09464 ± 0.03830; 95% CI = −0.1748 to −0.01448	9 LMs (4F, 5M); mean, 0.2139; 12 KOs (7F, 5M) mean, 0.1192	Breeding generations F3–F4. Some animals have been excluded due to poor quality of vGluT1 staining
Figure 4*F* (overlapping CB1 and vGluT1)	Normal distribution. *F* test to compare variances, *F*, DFn, Dfd = 1.758, 8, 11	Unpaired *T* test	*P* value of unpaired *T* test, 0.0109; the difference between means ± SEM = −5,374 ± 1,905; 95% CI = −9,362 to −1,387	9 LMs (4F, 5M); mean, 11,848 μm^3^ overlap volume; 12 (7F, 5M) KOs; mean, 6,474 μm^3^ overlap volume	Breeding generations F3–F4. Some animals have been excluded due to poor quality of vGluT1 staining
Figure 4*G* (ratio of overlapping over vGluT1)	Normal distribution. *F* test to compare variances, *F*, DFn, Dfd = 1.069, 8, 11	Unpaired *T* test	*P* value of unpaired *T* test, 0.0214; the difference between means ± SEM = −0.1841 ± 0.07341; 95% CI = −0.3377 to −0.03041	9 LMs (4F, 5M); mean, 0.4158; 12 (7F, 5M) KOs; mean, 0.2318	Breeding generations F3–F4. Some animals have been excluded due to poor quality of vGluT1 staining
Figure 6*B* (horizontal ladder locomotion)	Normal distribution. *F* test to compare variances, *F*, DFn, Dfd = 1.086, 23, 22	Unpaired *T* test	The *p* value of unpaired *T* test, 0.0091; the difference between means ± SEM = −6.520 ± 2.393; 95% CI = −11.34 to −1.700	24 LMs (9 females + 15 males); mean, 16.95% slips; 23 KOs (12 females + 11 males); mean, 10.43% slips	Breeding generations F4
Figure 6*C* (total time to cross the ladder)	Normal distribution. *F* test to compare variances, *F*, DFn, Dfd = 1.762, 20, 22	Unpaired *T* test	*P* value of unpaired *T* test, 0.9545; the difference between means ± SEM = −0.2754 ± 4.793; 95% CI = −9.948 to 9.398	23 LMs (9 females + 14 males); mean, 22.61 s; 21 KOs (10 females + 11 males); mean, 22.33 s	Breeding generations F4; same mice as in Figure 6*B*. 1 LM and 2 KOs have been excluded due to switching directions while walking on the ladder, so that their total time to cross is not comparable to their peers
Figure 7*A* (millet seed retrieval)	Nonlinear fit	Timecourse, area under the curve, nonlinear fit	LMs area under the curve, 541.4; CI, 490.6–592.2; slope, 0.8444–3.4117; *R*^2^ = 0.1299; KOs area under the curve, 474.1; CI, 403.0–545.2; slope, 0.5756–3.099; *R*^2^ = 0.1705	9 LMs (5 females + 5 males); 7 KOs (2 females + 5 males)	Breeding generation F3
Figure 7*B* (millet seed retrieval last day of testing and retesting)	Ordinary two-way ANOVA. Do not assume sphericity (equal variability of differences)	2-way ANOVA (same animal tested at two timepoints: on the last day of testing and retesting 1 month later)	Last day of testing: *p* value of 2-way ANOVA KO and LM = 0.7389; retesting *p* value of 2-way ANOVA KO and LM, 0.1434; *p* value of 2-way ANOVA last day of testing vs re testing, >0.0001. Overall difference between LM and KO: mean of LM, 41.90; mean of KO, 47.14; difference between means, −5.243; SE of difference, 3.396; 95% CI of difference, −12.48 to 1.995. Overall difference between testing and retesting: mean of last day of testing, 58.94; mean of retesting, 30.10; difference between means, 28.84; SE of difference, 4.019; 95% CI of difference, 20.28–37.41. Interaction CI: Mean diff, A1 − B1 −0.6857 Mean diff, A2 − B2 −9.800 (A1 − B1) − (A2 − B2) 9.114 95% CI of difference, −8.017 to 26.25 (B1 − A1) − (B2 − A2) −9.114 95% CI of difference, −26.25 to 8.017	Last day of testing: 10 LMs (5 females + 5 males); mean, 58.60%; 7 KOs (2 females + 5 males); mean, 59.29%; retesting: 10 LMs (5 females + 5 males); mean, 25.20%; 7 KOs (2 females + 5 males); mean, 35.00%	Breeding generation F3; same mice as in Figure 7*A*
Figure 6*E* (preference to explore social cues)	Normal distribution. *F* test to compare variances, *F*, DFn, Dfd = 1.577, 25, 23	Unpaired *T* test	*P* value of unpaired *T* test, 0.002; the difference between means ± SEM = −0.7570 ± 0.2310; 95% CI, −1.221 to −0.2926	26 LMs (13 females + 13 males); mean, 2.402; 24 KOs (13 females + 11 males); mean, 1.645	Breeding generation F4
Figure 6*F* (overall locomotion in social olfactory preference 3-chamber test)	Normal distribution. *F* test to compare variances, *F*, DFn, Dfd = 1.122, 22, 16	Unpaired *T* test	*P* value of unpaired *T* test, 0.0705; the difference between means ± SEM = 453.3 ± 243.6; 95% CI, −39.80 to 946.3	17 LMs (10 females + 7 males); mean, 40.31 m; 23 KOs (13 females + 10 males); mean, 44.85 m	Breeding generation F4. Same mice as in Figure 6. 3 LM females and 6 LM males were excluded after quality control of EthoVision XT 17 (Noldus) automated data analysis. In the affected videos, the setup of the chamber for that testing day resulted in a shadow in the arena, which interfered with total locomotion analysis
Figure 8*A*. Paired analysis of cumulative duration in the zone: (S) cup vs (N) cup	LM: correlation coefficient *R* = 0.3466; *p* value (one tailed) = 0.0414. The pairing was significantly effective; KO: correlation coefficient *R* = 0.3466, *p* value (one tailed) = 0.0414. The pairing significantly effective	Paired *T* test	LM: *p* value of paired *T* test, <0.0001. Mean of differences (B − A), −53.45. SD of differences, 35.41. SEM of differences, 6.945. 95% CI, −67.75 to −39.15. *R*^2^ = 0.7032; KO: *p* value of paired *T* test, 0.0312. Mean of differences (B − A), −19.85. SD of differences, 41.37. SEM of differences, 8.627. 95% CI, −37.75 to −1.964. *R*^2^ = 0.1941. Unpaired *T* test of cumulative durations in (S) for LMs and KO: *p* = 0.008; unpaired *T* test of cumulative durations in (N) for LMs and KOs *p* = 0.2293	26 LMs (13 females + 13 males), 23 KOs (13 females + 10 males)	Breeding generation F4. Same mice as in Figure 6
Figure 8*B*. ANOVA of cumulative duration in (S) and (N) zones LM vs KO	Ordinary two-way ANOVA. Do not assume sphericity (equal variability of differences)	2-way ANOVA (same animal tested at (S) and (N) conditions)	LM vs KO at (S) cup: *p* = 0.0099; difference between means ± SEM = −24.66 ± 9.168; 95% CI of difference, −43.11 to −6.221; LM vs KO at (N) cup: *p* = 0.2247; difference between means ± SEM = 8.198 ± 6.663; 95% CI of difference, −5.206 to 21.60; females LM vs KO (S) cup: *p* = 0.0693; difference between means ± SEM = −23.23 ± 12.22; 95% CI of difference, −48.45 to 1.983; females LM vs KO (N) cup: *p* = 0.0072; difference between means ± SEM = 18.27 ± 6.220; 95% CI of difference, 5.430–31.11; males LM vs KO (S) cup: *p* = 0.0715; difference between means ± SEM = −27.20 ± 14.33; 95% CI of difference, −57.01 to 2.601; males LM vs KO (N) cup: *p* = 0.8320; difference between means ± SEM = −2.627 ± 12.23; 95% CI of difference, −28.06 to 22.81	27 LMs (13 females + 13 males), 23 KOs (13 females + 10 males)	Breeding generation F4. Same mice as in Figure 6
Figure 8*C*. Heatmaps of cumulative duration in (S) and (N) zones		Cumulative time in *X*, *Y* coordinates; scales 0 (blue) to 50 (red)	Output from EthoVision XT 17	28 LMs (13 females + 13 males), 23 KOs (13 females + 10 males)	Breeding generation F4. Same mice as in Figure 6
Figure 6*H* (open-field center time)	Normal distribution. *F* test to compare variances, *F*, DFn, Dfd = 1.166, 21, 15	Unpaired *T* test	*P* value of unpaired *T* test, 0.0057; the difference between means ± SEM = −28.97 ± 9.844; 95% CI, −48.93 to −9.003	22 LMs (13 females + 9 males); mean, 88.18 s; 16 KOs (9 females + 7 males); mean, 59.21 s	Breeding generation F4
Figure 6*I* (total locomotion in open field)	Normal distribution. *F* test to compare variances, *F*, DFn, Dfd = 1.060, 22, 15	Unpaired *T* test	*P* value of unpaired *T* test, 0.2008; the difference between means ± SEM = −3.545 ± 2.722; 95% CI, −9.060 to 1.969	22 LMs (13 females + 9 males); mean, 46.09 m, 16 KOs (9 females + 7 males); mean, 42.54 m	Breeding generation F4. Same mice as in Figure 6*H*
Figure 7*C* (predator smell fear immobility)	Ordinary two-way ANOVA. Do not assume sphericity (equal variability of differences)	2-way ANOVA (same animal tested at two conditions: water and TMT)	Water LM vs KO: *p* = 0.3638; difference between means ± SEM = 2.050 ± 2.232; 95% CI of difference, −2.455 to 6.555; TMT LM vs KO: *p* = 0.6095; difference between means ± SEM = −2.205 ± 4.285; 95% CI of difference, −10.85 to 6.442; LMs water vs TMT: *p* = <0.0001; difference between means ± SEM = 20.65 ± 2.903; 95% CI of difference, 14.81–26.50; KOs water vs TMT: *p* = 0.0002; difference between means ± SEM = 16.40 ± 3.987; 95% CI of difference, 8.327 to 24.47. Interaction CI: Mean diff, A1 − B1 −2.050 Mean diff, A2 − B2 2.205 (A1 − B1) − (A2 − B2) −4.255 95% CI of difference, −12.48 to 3.972 (B1 − A1) − (B2 − A2) 4.255 95% CI of difference, −3.972 to 12.48	Water: 24 LMs (9 females, 15 males); mean, 5.352; 20 KOs (12 females, 8 males); mean, 7.412; TMT: LMs mean, 26.02; 20 mean, 23.81	Breeding generation F4
Figure 7*D* (grooming)	*F* test to compare variances, *F*, DFn, Dfd = 2.400, 19, 23. The variance between groups is significantly different (*p* = 0.0473), with the distribution of values tighter in LMs as compared with KOs	Unpaired *T* test	*P* value of unpaired *T* test, 0.8499; the difference between means ± SEM = 0.4853 ± 2.548; 95% CI, −4.657 to 5.627	24 LMs (9 females + 15 males); mean, 14.91; 20 KOs (12 females + 8 males); mean, 15.39	Breeding generation F4. Same mice as in Figure 7*C*
Figure 7*E* (marbles buried)	Normal distribution. *F* test to compare variances, *F*, DFn, Dfd = 1.140, 9, 17	Unpaired *T* test	*P* value of unpaired *T* test, 0.7938; the difference between means ± SEM = −3.278 ± 12.41; 95% CI, −28.79 to 22.23	18 LMs (8 females + 10 males); mean, 40.78; 10 KOs (5 females + 5 males); mean, 37.50	Breeding generation F3
Figure 7*F* (impulsive reaches)	Ordinary tow-way ANOVA. Do not assume sphericity (equal variability of differences)	2-way ANOVA (same animal tested at two timepoints: on the last day of testing and retesting 1 month later)	Last day of testing: *p* value of unpaired *T* test, 0.1315; the difference between means ± SEM = −10.40 ± 6.492; 95% CI, −24.32 to 3.523; 1 month later: *p* value of unpaired *T* test, 0.9969; the difference between means ± SEM = −0.03175 ± 8.025; 95% CI, −17.24 to 17.18. Interaction CI: mean diff, A1 − B1 10.40 Mean diff, A2 − B2 0.03175 (A1 − B1) − (A2 − B2) 10.37 95% CI of difference, −10.83 to 31.57 (B1 − A1) − (B2 − A2) −10.37 95% CI of difference, −31.57 to 10.83	Last day of testing: 10 LMs (5 females + 5 males); mean, 23.40; 7 KOs (2 females + 5 males); mean, 13.00; retesting 1 month later: LM mean, 29.89; KO mean, 29.86	Breeding generation F3; same mice as in Figure 7*A*
Figure 9*G* (vGluT1 puncta on PC dendritic and somatic compartments)	Normal distribution. On dendrites: *F* test to compare variances, *F*, DFn, Dfd = 1.017, 11, 7. On soma: *F* test to compare variances, *F*, DFn, Dfd = 2.988, 7, 11	Unpaired *T* test	On dendrites: *p* value of unpaired *T* test, 0.7828; the difference between means ± SEM = −0.007363 ± 0.02631; 95% CI, −0.06264 to 0.04792. On soma: *p* value of unpaired *T* test, 0.2088; the difference between means ± SEM = −0.02136 ± 0.01638; 95% CI, −0.05577 to 0.01306	8 LMs (4 females + 4 males); mean on dendrite, 0.2290; mean on soma, 0.06399; 12 KOs (7 females + 5 males); mean on dendrite, 0.2216; mean on soma, 0.04263	Breeding generations F3 and F4; Same mice as in Figure 4. Some animals have been excluded due to poor quality of vGluT1 staining
Figure 9*H* (vGluT2 puncta on PC dendritic and somatic compartments)	Normal distribution. On dendrites: *F* test to compare variances, *F*, DFn, Dfd = 1.060, 15, 13. On soma: *F* test to compare variances, *F*, DFn, Dfd = 1.413, 15, 13	Unpaired *T* test	On dendrites: *p* value of unpaired *T* test, 0.4206; the difference between means ± SEM = −0.0003294 ± 0.0004030; 95% CI, −0.001155 to 0.0004960. On soma: *p* value of unpaired *T* test, 0.6579; the difference between means ± SEM = 0.0002533 ± 0.0005658; 95% CI, −0.0009057 to 0.001412	9 LMs (4 females + 4 males); mean on dendrite, 0.004802; mean on soma, 0.002124; 12 KOs (7 females + 5 males); mean on dendrite, 0.004473; mean on soma, 0.002377	Breeding generations F3 and F4; same mice as in Figure 4
Figure 9*I* (vGaT puncta on PC dendritic and somatic compartments)	Normal distribution. On dendrites: *F* test to compare variances, *F*, DFn, Dfd = 1.260, 15, 13. On soma: *F* test to compare variances, *F*, DFn, Dfd = 2.053, 13, 15	Unpaired *T* test	On dendrites: *p* value of unpaired *T* test = 0.6250; the difference between means ± SEM = 0.001891 ± 0.003825; 95% CI, −0.005945 to 0.009726. On soma: *p* value of unpaired *T* test, 0.2189; the difference between means ± SEM = −0.01045 ± 0.008308; 95% CI, −0.02747 to 0.006569	10 LMs (4 females + 4 males); mean on dendrite, 0.03675; mean on soma, 0.06036; 12 KOs (7 females + 5 males); mean on dendrite, 0.03864; mean on soma, 0.04991	Breeding generations F3 and F4; Same mice as in Figure 4
Figure 10 (sEPSCs and sIPSCs)	Normal distribution. sEPSC amp: *F* test to compare variances, *F*, DFn, Dfd = 2.772, 8, 10. sEPSC freq: *F* test to compare variances, *F*, DFn, Dfd = 1.434, 10, 8. sEPSC rise: *F* test to compare variances, *F*, DFn, Dfd = 1.095, 10, 8. sEPSC tau: *F* test to compare variances, *F*, DFn, Dfd = 1.650, 10, 8. sIPSC amp: *F* test to compare variances, *F*, DFn, Dfd = 2.439, 8, 8. sIPSC freq: *F* test to compare variances, *F*, DFn, Dfd = 1.425, 8, 8. sIPSC rise: *F* test to compare variances, *F*, DFn, Dfd = 2.078, 8, 8. sIPSC tau: *F* test to compare variances, *F*, DFn, Dfd = 1.041, 8, 8	Unpaired *T* test	sEPSC amp: *p* value of unpaired *T* test, 0.193; the difference between means ± SEM = −1.529 ± 1.131; 95% CI, −3.906 to 0.8467. sEPSC freq: *p* value of unpaired *T* test, 0.3254; the difference between means ± SEM = −1.013 ± 1.001; 95% CI, −3.117 to 1.091. sEPSC rise: *p* value of unpaired *T* test, 0.2797; the difference between means ± SEM = −0.8551 ± 0.7672; 95% CI, −2.467 to 0.7568. sEPSC tau: *p* value of unpaired *T* test, 0.9092; the difference between means ± SEM = −0.7003 ± 6.058; 95% CI, −13.43 to 12.03. sIPSC amp: *p* value of unpaired *T* test, 0.9429; the difference between means ± SEM = −0.1739 ± 2.389; 95% CI, −5.238 to 4.890. sIPSC freq: *p* value of unpaired *T* test, 0.7624; the difference between means ± SEM = 0.5886 ± 1.914; 95% CI, −3.469 to 4.646. sIPSC rise: *p* value of unpaired *T* test, 0.9063; the difference between means ± SEM = 0.1111 ± 0.9287; 95% CI, −1.858 to 2.080. sIPSC tau: *p* value of unpaired *T* test, 0.2654; the difference between means ± SEM = −4.586 ± 3.974; 95% CI, −13.01 to 3.838	sEPSC: 9 LM, 11 KO. sIPSC: 9 LM, 9 KO	Breeding generations F4 and F5
Figure 11*C* (DSE)	Normal distribution. *F* test to compare variances, *F*, DFn, Dfd = 1.300, 6, 8	Unpaired *T* test	Cumulative amplitude ratio: *p* value of unpaired *T* test, 0.0065; the difference between means ± SEM = 0.3330 ± 0.1042; 95% CI, 0.1094–0.5565	7 LMs mean, 0.4149; 9 KOs mean, 0.7478	Breeding generations F4 and F5
Figure 11*F* (DSI)	Normal distribution. *F* test to compare variances, *F*, DFn, Dfd = 2.555, 3, 8	Unpaired *T* test	Cumulative amplitude ratio: *p* value of unpaired *T* test, 0.0202; the difference between means ± SEM = 0.3770 ± 0.1389; 95% CI, 0.07124–0.6828	4 LMs mean, 0.5057; 9 KOs mean, 0.8996	Breeding generations F4 and F5
Figure 14*E* (cFos^+^ PCs per 100 μm in VI, VII)	Normal distribution. *F* test to compare variances, *F*, DFn, Dfd = 1.560, 13, 15	Unpaired *T* test	14 LMs mean, 18.08 cells per 100 μm; 16 KOs mean, 13.39 cells per 100 μm; a *p* value of unpaired *T* test, 0.0214; the difference between means ± SEM = −4.690 ± 1.924; 95% CI, −8.631 to −0.7488	14 LMs (7 females + 7 males); mean, 18.08 cells per 100 μm; 16 KOs (10 females + 6 males); mean, 13.39 cells per 100 μm	Breeding generations F3 and F4
Figure 14*F* (cFos^+^ PCs per 100 μm in dorsal VI)	Normal distribution. *F* test to compare variances, *F*, DFn, Dfd = 1.128, 13, 15	Unpaired *T* test	*P* value of unpaired *T* test, <0.0001; the difference between means ± SEM = −2.544 ± 0.3485; 95% CI, −3.258 to −1.830	14 LMs (7 females + 7 males); mean, 3.755 cells per 100 μm; 16 KOs (10 females + 6 males); mean, 1.210 cells per 100 μm	Breeding generations F3 and F4
Figure 14*G* (cFos^+^ MLIs per 10,000 μm^2^ in dorsal VI)	Normal distribution. *F* test to compare variances, *F*, DFn, Dfd = 1.035, 13, 15	Unpaired *T* test	*P* value of unpaired *T* test, 0.1574; the difference between means ± SEM = −1.042 ± 0.7171; 95% CI, −2.511 to 0.4272	14 LMs (7 females + 7 males); mean, 9.045 cells per 10,000 μm^2^; 16 KOs (10 females + 6 males); mean, 8.003 cells per 10,000 μm^2^	Breeding generations F3 and F4
Figure 14*H* (cFos^+^ GCs per 10,000 μm^2^ in dorsal VI)	Normal distribution. *F* test to compare variances, *F*, DFn, Dfd = 1.180, 13, 15	Unpaired *T* test	*P* value of unpaired *T* test, 0.1781; the difference between means ± SEM = −9.792 ± 7.089; 95% CI, −24.31 to 4.728	14 LMs (7 females + 7 males); mean, 52.66 cells per 10,000 μm^2^; 16 KOs (10 females + 6 males); mean, 42.86 cells per 10,000 μm^2^	Breeding generations F3 and F4
Figure 14*I* (correlation of c-Fos in PC vs social preference)	The correlation is not statistically significant	Correlation	95% CI LM, −0.7366 to 0.2345. 95% CI KO, −0.3598 to 0.6670. *R*^2^ LM = 0.1144, *R*^2^ KO = 0.04455	14 LMs (7 females + 7 males); Pearson's *r* LM = −0.3382. 14 KOs (8 females + 6 males); Pearson's *r* KO = 0.2111	Breeding generations F3 and F4

### Mouse behavior

Breeding generation F3 mice were used in millet seed retrieval and marble burying tests (the tests were administered in that order). Breeding generation F4 mice were used in horizontal ladder locomotion, social olfactory choice, open field, grooming, and predator smell immobility tests (the tests were administered in that order). Not all mice were used in every experiment—details are provided in the statistics table (Table 2).

#### Grooming (Fig. 7D)

Two-month-old mice were individually placed in a plexiglass testing chamber and video-recorded for 20 min. Mice have not been accommodated to the chamber prior to the 20 min video recording session. The videos were manually annotated from videos recorded at 60 fps for the percentage of time spent grooming. “Statistical analysis” was performed in GraphPad Prism 10 (www.graphpad.com). Breeding generation F4 mice (9 LM females, 15 LM males, 12 KO females, 8 KO males) were used. The statistical hypothesis tested the magnitude of differences between genotypes. An unpaired Student’s *T* test was used to evaluate means, SEMs, and *p* values.

#### Locomotion on the horizontal ladder with uneven rungs (Fig. 6B,C)

Two-month-old mice were placed on a horizontal ladder with unevenly spaced rungs. The ladder was placed between a clean mouse cage and the test mouse's home cage, and the mice were video-recorded at 60 fps from the ventral side as they walked across the ladder, allowing easy visibility of paw placement. The videos were manually scored frame by frame for the number of missteps and successful placements of hindpaws on the rungs. The percentage of missed steps (out of all steps) was calculated. “Statistical analysis” was performed in GraphPad Prism 10 (www.graphpad.com). Breeding generation F4 mice (9 LM females, 15 LM males, 12 KO females, 11 KO males) were used. The statistical hypothesis tested the magnitude of differences in the percentage of missteps and time to cross the ladder for KOs and LMs. An unpaired Student’s *T* test was used to evaluate means, SEMs, and *p* values. Estimation plots were used to analyze the CIs for the differences between means.

#### Marble burying (Fig. 7E)

Two-month-old mice were placed in a clean standard mouse cage filled with 10 cups of corn cob bedding (∼5 cm deep) for 15 min to habituate. After habituation, the corn cob bedding in the cage was smoothed down, and eight evenly spaced marbles were placed on top of the corn cob bedding. The mouse was placed back in the test cage and allowed to explore *ad libitum* for 15 min, after which the number of buried (≤1/4 visible) marbles was scored manually. “Statistical analysis” was performed in GraphPad Prism 10 (www.graphpad.com). Breeding generation F3 mice (8 LM females, 10 LM males, 5 KO females, 5 KO males) were used. The statistical hypothesis tested the magnitude of differences in the percentage of marbles buried between KOs and LMs. An unpaired Student’s *T* test was used to evaluate means, SEMs, and *p* values. A total of 107 animals were used in microscopy studies.

#### Millet seed reaching and retrieval; impulsive reaching (Fig. 7A,B,F)

To motivate the learning of a new skilled reaching task—extending a forelimb through a narrow slit to reach for, grasp, and retrieve a millet seed—we food-restricted the 2-month-old mice starting 3 d before and for the duration of the experiment. The weight of the mice was monitored daily, making sure that no individual lost >10% of body weight. Every morning, regular mouse chow was added to the cages, estimating the amount of food per day by adding the weight of the chow that corresponds to 10% of the collective weight of the mice in the cage (i.e., if the mice in the cage weighed 100 g, 10 g of chow was added to the cage once a day). Mice were placed one by one in a plexiglass behavior box from which they reached out through a slit with their forepaw to retrieve a millet seed. The experiment started with 3 d of training, during which the mice learned the task. Starting on the fourth day or after the mice exhibited proficiency in performing the task (being able to retrieve at least 10 seeds in 20 min and exhibiting consistent forepaw preference), the mice were placed in the apparatus for 20 min each day for 10 additional days, while seed reaching was video-recorded at 60 fps. The resulting videos were manually scored for the percentage of successfully retrieved millet seeds out of all the reaching attempts with the seeds present. Millet seeds were added to the tray in front of the slit one by one, and the mice would occasionally reach through the slit even when there were no seeds present on the tray (impulsive reaching). “Statistical analysis” was performed in GraphPad Prism 10 (www.graphpad.com). Breeding generation F3 mice (five LM females, five LM males, two KO females, five KO males) were used. The graph shows the percentage of successfully retrieved seeds on each day for a 10 d timecourse. The data are shown as means + SD. Areas under the curve and CIs were assessed for the learning timecourse graphs and showed no differences between KOs and LMs. The percentage of successfully retrieved seeds was evaluated on the last day of testing and 1 month after the last day of testing. The statistical hypothesis tested the magnitude of differences in the percentage of successfully retrieved seeds (out of all attempts where seeds were present on the tray) and the percentage of impulsive reaches (out of all reaches) for the last day of testing and the 30 d later retesting between KOs and LMs. Two-way ANOVA was used.

#### Open field (Fig. 6H,I)

Two-month-old mice were placed in an open plexiglass arena for 10 min, and their movement was video-recorded using Pylon Viewer and EthoVision XT 17. The amount of time spent in the center of the arena and the total distance moved in the arena were calculated. “Statistical analysis” was performed in GraphPad Prism 10 (www.graphpad.com). Breeding generation F4 mice (13 LM females, 9 LM males, 9 KO females, 7 KO males) were used. The statistical hypothesis tested the magnitude of differences in cumulative time spent in the center of the arena and cumulative distance explored between KOs and LMs. An unpaired Student’s *T* test was used to evaluate means, SEMs, and *p* values. Estimation plots were used to analyze the CIs for the differences between means.

#### Predator fear-induced immobility (Fig. 7C)

Two-month-old mice were placed in a plexiglass testing chamber for 20 min and video-recorded at 60 fps. On Day 1, the mice were exposed to 10 μl of distilled water pipetted onto a cotton ball inside a 20 ml uncapped scintillation vial suspended out of reach inside the testing chamber. On the next day, the experiment was repeated in the same chamber, but the water was replaced with 10 μl of 2,5-dihydro-2,4,5-trimethylthiazoline (TMT—an aromatic compound from fox urine). The videos were analyzed with ezTrack (Pennington et al., 2019) to determine the percentage of time spent immobile. “Statistical analysis” was performed in GraphPad Prism 10 (www.graphpad.com). Breeding generation F4 mice (9 LM females, 15 LM males, 12 KO females, 8 KO males) were used. The statistical hypothesis tested the magnitude of differences between genotypes in the percentage of time spent immobile during exposure to water and TMT. Paired analysis shows significantly increased freezing for TMT compared with water for both genotypes but no differences between the genotypes. Two-way ANOVA was used.

#### Social olfactory exploration (Figs. 6E,F, 8)

Two-month-old mice were individually placed in a three-chamber plexiglass arena with two wire cups covering 35 mm Petri dishes containing neutral (N; i.e., clean) bedding. The mice were allowed to explore the arena with the two N dishes for 10 min. Subsequently, one dish was replaced with a social bedding dish (S; i.e., soiled bedding from a cage of sex- and age-matched unfamiliar mice), and the mice explored the arena for another 10 min while video-recorded using Pylon Viewer and EthoVision XT 17. The arena was thoroughly cleaned between mice, and the side (right, R or left, L) of the (S) cup placement was alternated for all trials in the sequence. The frequency of nose pokes (to sniff) at the neutral and the social cups was scored manually, and the ratio of social versus neutral nose pokes was calculated. Cumulative durations spent exploring (S) and (N) cups, heatmaps, and total distance moved were assessed automatically in EthoVision XT 17. “Statistical analysis” was performed in GraphPad Prism 10 (www.graphpad.com). Breeding generation F4 mice (13 LM females, 13 LM males, 13 KO females, 10 KO males) were used to assess the ratio of social over neutral nose pokes. For determining the total distance traveled, since shadows in the arena interfered with automatic tracking for a few animals, three LM females and six LM males were excluded from the analysis. The statistical hypothesis tested the magnitude of differences in the frequency of social over neutral nose pokes and the total distance moved between KOs and LMs. An unpaired Student’s *T* test was used to evaluate means, SEMs, and *p* values. Estimation plots were used to analyze the CIs for the differences between means.

#### Electrophysiology (Figs. 10–12)

Postnatal day (P)25–31 mice were anesthetized with isoflurane. The brains were carefully and promptly removed and blocked. Parasagittal 280-μm-thick slices of the cerebellum were cut at 0.1 mm/s with a Leica VT1200 vibratome in ice-cold oxygenated external solution containing a sucrose-based artificial cerebrospinal fluid (aCSF; in mM: 194 sucrose, 30 NaCl, 26 NaHCO_3_, 10 glucose, 4.5 KCl, 0.5 NaH_2_PO_4_, 1 MgCl_2_), pH 7.4, bubbled with 95% O_2_/5% CO_2_. After cutting, slices were transferred to an incubation chamber containing oxygenated aCSF solution at 33°C for 1 h. Slices were then kept at room temperature until recording.

Whole-cell patch recordings were done from PC identified by TOM fluorescence at 30–32°C in a submersion chamber perfused (∼2 ml/min) with aCSF solution comprised of the following (in mM): 124 NaCl, 4.5 KCl, 1.2 NaH_2_PO_4_, 1 MgCl_2_, 2 CaCl_2_, 26 NaHCO_3_, and 10 glucose, continuously bubbled with 95% O_2_/5% CO_2_, pH 7.4, 310 mOsm. Patch pipettes (2–3 MΩ) were pulled from borosilicate glass and filled with internal solution containing the following (in mM): 120 CsMeSO_3_; 5 NaCl, 10 TEA, 10 HEPES, 5 lidocaine bromide, 1.1 EGTA, 4 Mg-ATP, and 0.3 Na-GTP and 0.2% biocytin, pH 7.3 (292 mOsm), for voltage-clamp recordings. Biocytin (2 mg/ml) was also added to the internal solution to allow for post hoc visualization of recorded neurons. TOM-positive neurons were identified and visualized with a 40× water-immersion objective on an upright fluorescent microscope (BX51WI; Olympus) equipped with infrared-differential interference contrast video microscopy and epifluorescence through the light path of the microscope using an ultrahigh-powered light–emitting diode (CoolLED). Data were collected using the Clampex software (version 10, Molecular Devices). Signals were filtered using a Bessel filter set at 4 kHz and digitized at 50 kHz with a Digidata 1440A A/D interface. A 5 mV pulse was delivered regularly to monitor series resistance. Recordings with series resistance >25 MΩ or with changes in access >20% were discarded. For quantification, the software pClamp11 (Molecular Devices) and MiniAnalysis (Synaptosoft) were used. Statistical analysis was done using Prism 10 (GraphPad Software). The holding membrane potential was set to −70 mV in all experiments.

Spontaneous excitatory postsynaptic currents (sEPSCs) were recorded after adding picrotoxin (50 µM) to the aCSF to block inhibitory currents (nine CONT PCs, eight KO PCs). Spontaneous inhibitory PSCs (sIPSCs) were recorded after adding NBQX (10 µM) and D-AP5 (50 µM) to block excitatory currents (nine CONT PCs, eight KO PCs). Amplitude, Frequency, rise time, and decay time (tau) were measured. The holding membrane potential was (*V_h_*) at −70 mV (nine CONT PCs, eight KO PCs). For evoked PSCs, a stimulating bipolar electrode was placed in the ML, and synaptic responses were evoked at 0.2 Hz (rectangular pulses 0.2 ms duration, 50–300 μA).

To induce DSE and DSI, a depolarizing pulse from −70 to 0 mV was applied once for 5 s after 2 min of baseline recording. Excitatory and inhibitory currents were isolated, as was done in sEPSC and sIPSC recordings described above.

Additional details on the number of neurons recorded and the animals used for electrophysiology experiments are reported in Table 3.

**Table 3.** Neurons and mice used in electrophysiology experiments

1	LM Mouse 1	3 cells	3 sEPSC	3 DSE	LM
2	LM Mouse 2	4 cells	4 sEPSC	2 DSE	LM
3	LM Mouse 3	3 cells	2 sEPSC + 1 sIPSC	2 DSE	LM
4	LM Mouse 4	5 cells	5 sIPSC	2 DSI	LM
5	LM Mouse 5	3 cells	3 sIPSC	2 DSI	LM
6	KO Mouse 1	1 cell	1 sEPSC	1 DSE	KO
7	KO Mouse 2	4 cells	4 sEPSC	3 DSE	KO
8	KO Mouse 3	2 cells	2 sIPSC	2 DSI	KO
9	KO Mouse 4	4 cells	4 sIPSC	3 DSI	KO
10	KO Mouse 5	3 cells	3 sEPSC	3 DSE	KO
11	KO Mouse 6	1 cell	1 sIPSC	1 DSI	KO
12	KO Mouse 7	3 cells	2 sEPSC + 1 sIPSC	2 DSE + 1 DSI	KO
13	KO Mouse 8	2 cells	1 sEPSC + 1 sIPSC	1 DSE + 1 DSI	KO

A total of 284 animals were used in all studies combined.

## Results

### Daglα is selectively deleted from postnatal cerebellar PCs utilizing a Pcp2-Cre mouse line

We generated a PC-specific Daglα KO mouse line by crossing the conditional floxed *Daglα* (*Daglα^fl^*) mouse generated by the group of Dr. Sachin Patel (Bluett et al., 2017) with PC-specific Cre mouse line (*Pcp2^Cre^*) generated by the group of Noboru Suzuki (Saito et al., 2005). TOM reporter (*Ai9^f/f^*, The Jackson Laboratory, https://www.jax.org/strain/007909) was included in the breeding strategy to facilitate the identification of the recombined cells. LM controls (genotype *Pcp2^Cre/Cre^; Ai9^f/f^; Daglα^WT/WT^*) and conditional KOs (genotype *Pcp2^Cre/Cre^; Ai9^f/f^; Daglα^fl/fl^*) were generated by breeding males and females heterozygous for *Daglα^fl^* alle (*Pcp2^Cre/Cre^; Ai9^f/f^; Daglα^fl/WT^*; Fig. 1*A*). We confirmed the specificity and selectivity of Pcp2^Cre^ recombination in the adult mouse brain (Fig. 1*B*,*B*’, red showing TOM reporter expression in cerebellar PCs) in agreement with our prior characterization of this Pcp2^Cre^ mouse line (Kalinovsky et al., 2011).

**Figure 2.**
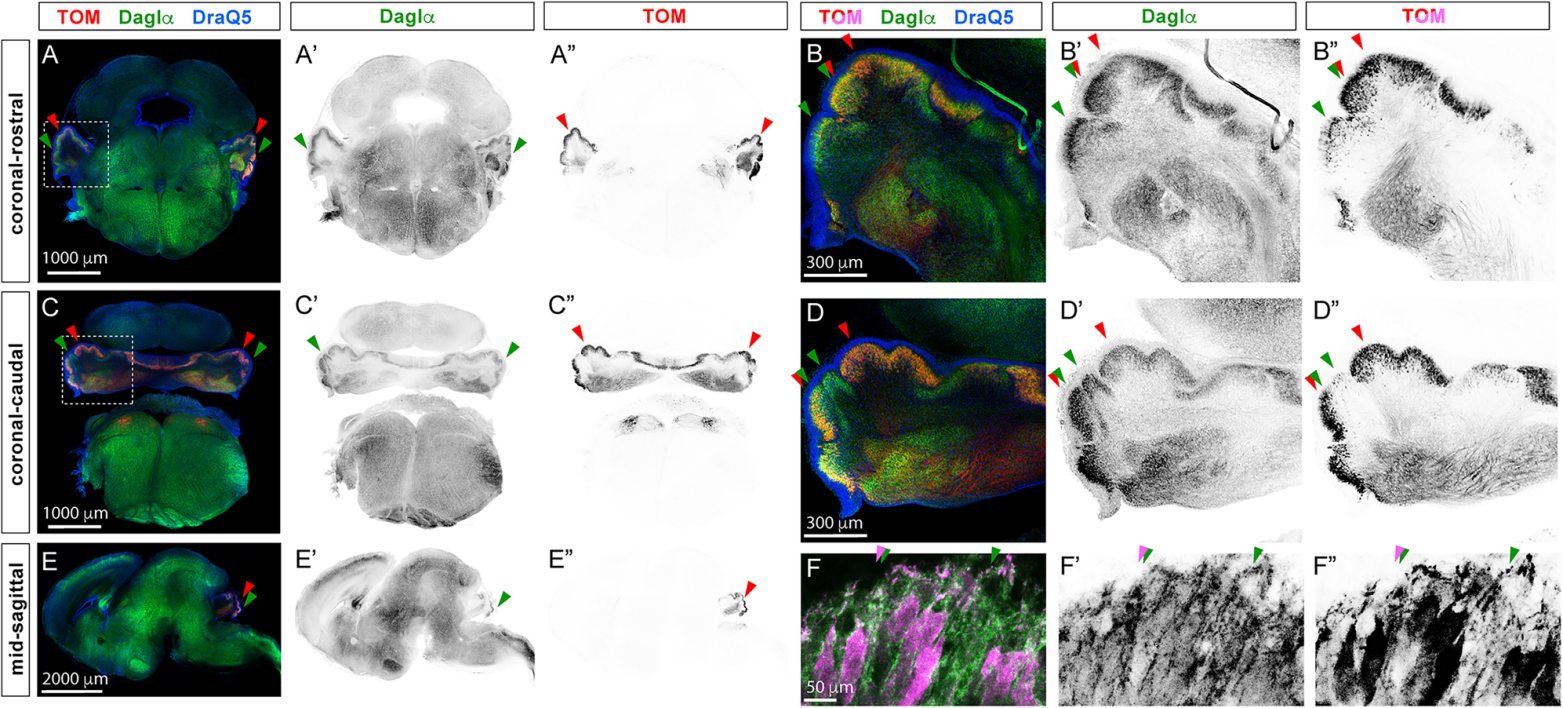
Pcp2^Cre^ recombination is efficient at birth. ***A*–*B*”**, A representative coronal–anterior section from a LM (Pcp2^Cre/Cre^; Ai9^fl/fl^; DAGLα^WT/WT^) at the day of birth (P0). The green arrows highlight the prominent expression of Daglα in the cerebellar PCs, and red arrows point to TOM reporter expression, verifying that Cre recombination is efficient and specific in the cerebellar PCs at birth. ***B*–*B*”**, A zoomed-in view of the cerebellum, showing that (***B*’**) the intensity of DAGLα expression varies across populations of PCs and (***B*”**) that a subpopulation of PCs is TOM-positive, indicating Pcp2-Cre–dependent recombination. ***C*–*D*”**, This pattern is consistent in coronal sections through a more caudal aspect of the cerebellar vermis. ***E*–*E*”**, A midsagittal section from a P0 mouse highlighting (***E*’**) the wide expression of DAGLα throughout the brain and (***E*”**) the very restricted TOM expression in cerebellar PCs. ***F*–*F*”**, Higher magnification of the cerebellum showing the multiple cell layer of PCs, typical for P0. ***F*’**, DAGLα is expressed in the plasma membrane of PCs in somata and dendrites, while (***F*”**) cytosolic TOM fills PCs. However, DAGLα and TOM clearly colocalize in some cells, while other cells are DAGLα-positive but TOM-negative. Eight LMs, 2F, 2M, sectioned sagittally, and 2F, 2M sectioned coronally.

At birth, Daglα is robustly expressed in the developing brain, with cerebellar expression primarily in PCs (Fig. *2A*,*A*’,*B*,*B*’,*C*,*C*’,*D*,*D*’,*E*,*E*’,*F*,*F*’, green arrowheads). Cre-dependent recombination is efficient and specific at birth, as indicated by TOM expression restricted to cerebellar PCs (Fig. 2*A*,*A*”,*B*,*B*”,*C*,*C*”,*D*,*D*”,*E*,*E*”,*F*,*F*”, red arrowheads). Zoomed-in coronal views of the cerebellum at both rostral and caudal levels show that the intensity of DAGLα expression varies across the populations of PCs (Fig. 2*B*,*B*’,*D*,*D*’) and that a subpopulation of PCs is TOM-positive, indicating Pcp2-Cre–dependent recombination (Fig. 2*B*,*B*”,*D*,*D*”,*E*–*E*”). A midsagittal view highlights the wide expression of DAGLα throughout the brain (Fig. 2*E*,*E*’) and the very restricted TOM expression in cerebellar PCs (Fig. 2*E*,*E*”). PCL in the sagittal section imaged with 60× objective shows DAGLα localization to somata and dendrites of PCs, while TOM expression is found in the cytoplasm of PC. DAGLα and Tom clearly colocalize in some PC; however, some DAGLα-positive PC are TOM-negative (Fig. 2*F*–*F*”). The patterns of Daglα and TOM expression were consistent between sexes and individual mice (eight LMs, 2F, 2M, sectioned sagittally; 2F, 2M sectioned coronally).

**Figure 3.**
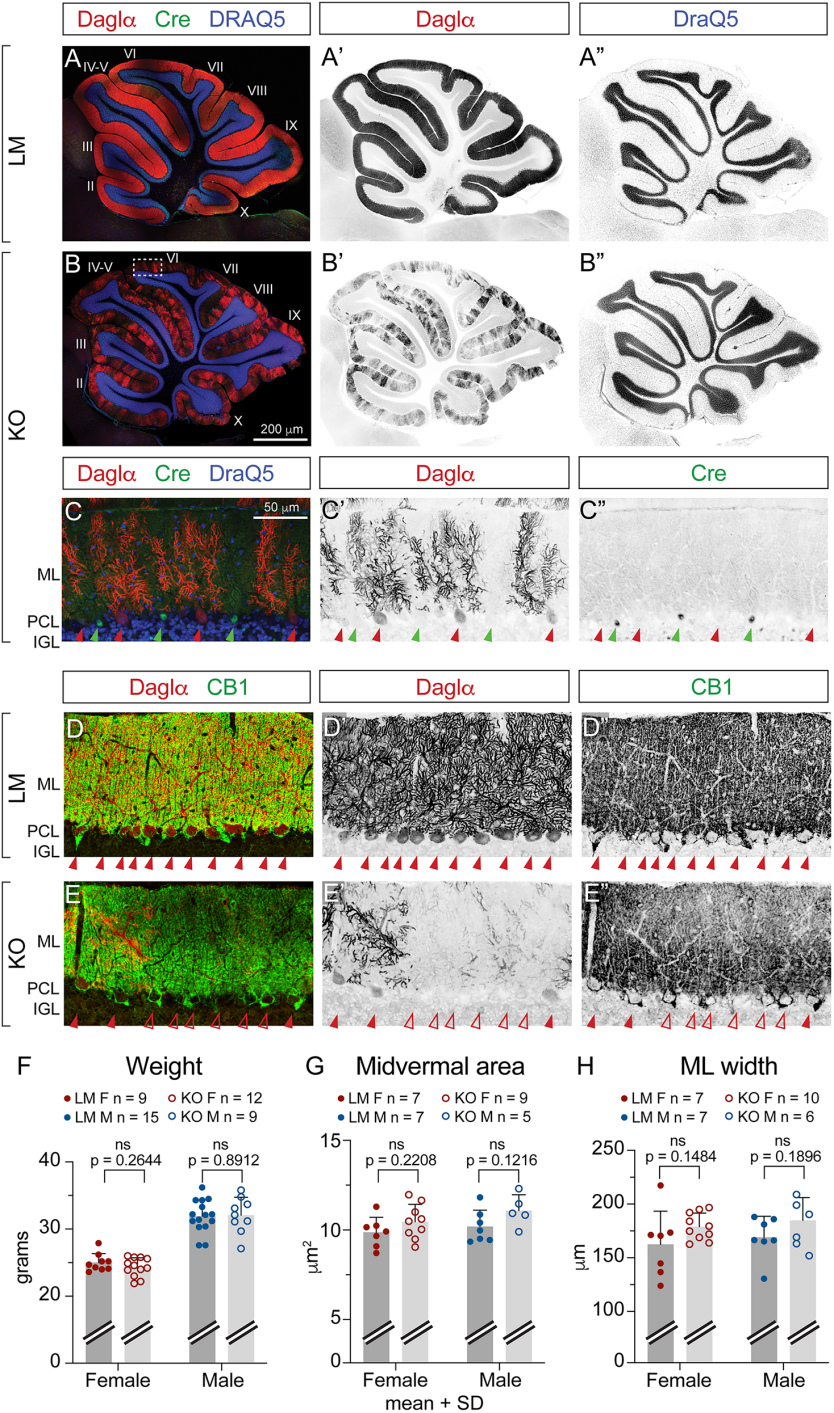
Daglα ablation is selective to Pcp2^Cre^-positive cerebellar PCs and does not affect cerebellar gross anatomy. Midsagittal sections from 2-month-old cerebella show that (***A***, ***A*’**) Daglα (red) is prominently expressed in the PCL in LMs. ***B***, ***B*’**, Daglα is lost in a subset of PCs in KOs. ***A*”**, ***B*”**, Nuclear DRAQ5 labeling (blue in ***A*** and ***B***) shows normal cerebellar vermis anatomy. ***C*–*C*”**, A higher-magnification view of lobe VI (dotted rectangle in ***B***) shows that Daglα expression is absent specifically in PCs that express Cre recombinase (green, highlighted by green arrowheads), while Cre-negative PCs continue to express Daglα, localized primarily to dendrites (ML) and soma (PCL). ***D*–*D*”**, A higher-magnification image of lobe VI showing Daglα (red) localized to somata and dendrites of PCs and CB1 (green) localized to the axons in the ML and to the perisomatic and pinceau synapses on PC somata and axon initial segments. ***E*–*E*”**, A lack of Daglα expression is evident in a subset of PCs in KOs. While the overall pattern of CB1 labeling is unchanged, the staining is dimmer in outer ML next to Daglα-negative PC dendrites. ***F***, The weights of KOs are normal at the age of 2 months. KOs exhibit no differences in anatomical parameters such as (***G***) the area of the cerebellar midvermis or (***H***) ML width. ML, molecular layer; PCL, PC layer; IGL, inner granule cell layer. Roman numerals designate the lobules of the cerebellar vermis. Columns show means + SD. An unpaired student *T* test was used to assess *p* values.

In the adult mouse cerebella, Daglα is highly expressed throughout the PCL (Fig. 3*A*,*A*’). Akin to other molecular markers of PCs, Pcp2^Cre^ is expressed in periodic domains along the PCL, and Cre-recombined PCs null for Daglα appear as striped patches in the KO (Fig. 3*B*,*B*’). Higher-magnification confocal microscopy with double immunostaining for Daglα (red) and Cre recombinase (green) confirms the specificity and selectivity of the Daglα KO from the Cre-positive PCs (Fig. 3*C*–*C*”). Daglα (red) subcellular localization to the somata and dendrites of PCs (Fig. 3*D*,*D*’) and the absence of Daglα expression in KO PCs (Fig. 3*E*,*E*’) are evident in 60× high-resolution images collected from the midvermal lobe VI. The major neuronal cannabinoid receptor, CB1 (green), is localized to the axons in the ML and to the presynaptic terminals of ML interneurons on PC somata and axon initial segments (perisomatic and pinceau synapses; Fig. 3*D*,*D*”). Less intense CB1 labeling in the outer ML next to Daglα-negative PC dendrites is evident in KOs (Fig. 3*E*,*E*”), suggesting reduced CB1 localization in parallel fiber (PF) synapses—as examined below.

### The overall physical development and anatomy are normal in KOs

Pcp2^Cre^-dependent KO of Daglα in PCs is induced around birth, allowing us to assess its requirements for the structural and functional postnatal development of cerebellar circuits. We conducted our anatomical analysis in 2-month-old young–adult mice at the endpoint of cerebellar neurocircuit development. Our empirical observations and the evaluation of body weights suggest that overall physical development is normal in KOs (Fig. 3*F*). Cerebellar anatomical parameters, such as total midvermal area and ML width, are comparable in KOs and LMs (Fig. 3*G*,*H*), suggesting that the numbers of cerebellar neurons and their localization into layers are unaffected by the loss of Daglα from PCs. In conclusion, our analysis revealed no abnormalities in the gross cerebellar anatomy in the KOs.

### CB1 localization is diminished in vGluT1-positive PF synapses in KOs

CB1 expression and localization can be regulated by ligand abundance. For example, depletion of synaptic CB1 is associated with elevated synaptic 2-AG levels in MAGL KOs (Schlosburg et al., 2010) or following exposure to exogenous CB1 agonists (Breivogel et al., 1999). Altered CB1 expression can, in turn, affect cannabinoid-dependent synaptic plasticity and neuronal sensitivity to exogenous cannabinoids. Previous studies demonstrated that this signaling intensity-dependent regulation of CB1 synaptic abundance is particularly robust in excitatory synapses (Ludányi et al., 2008).

**Figure 4.**
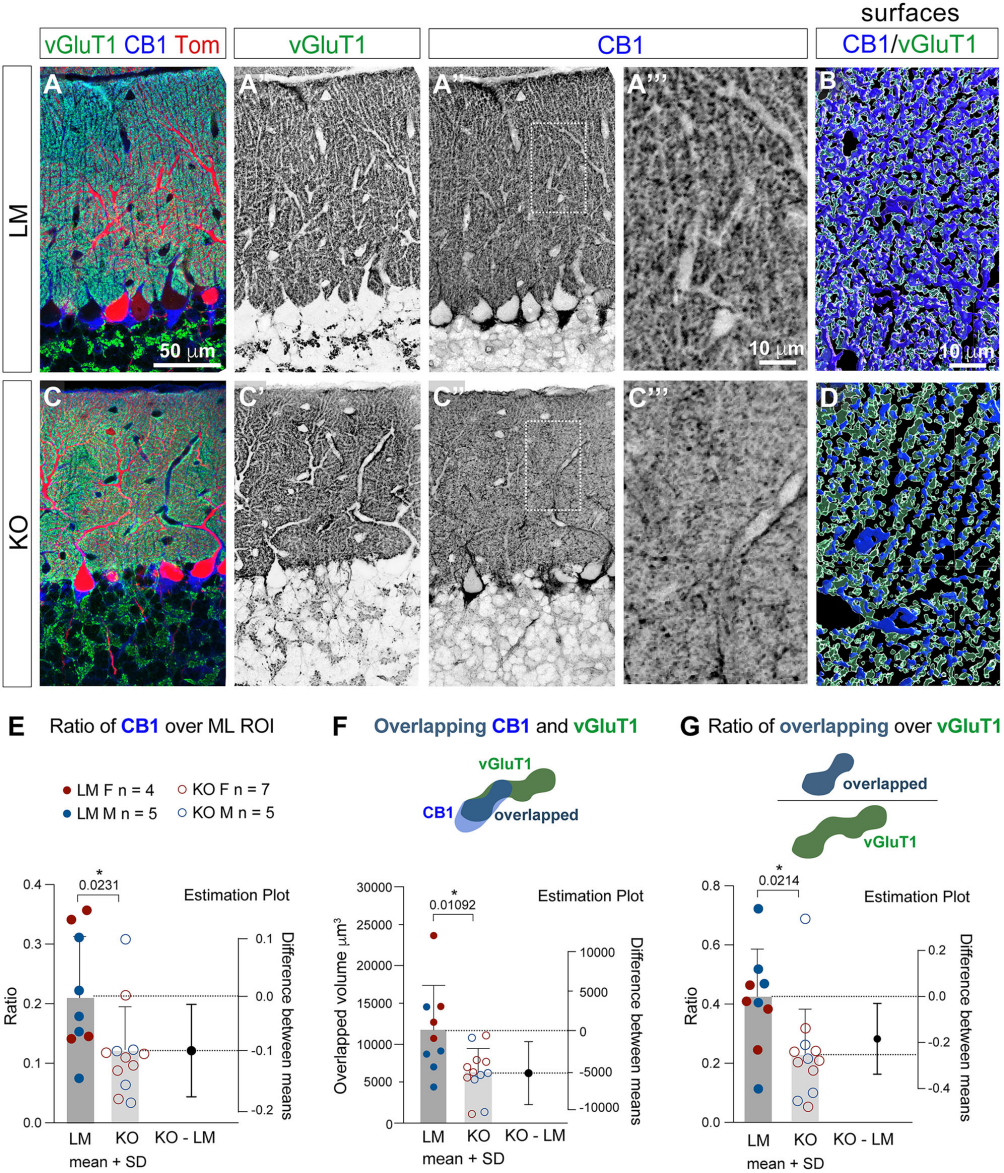
CB1 expression is reduced in presynaptic vGluT1-positive terminals between PF and Daglα KO PCs. ***A*–*B*’’’**, High-resolution confocal stacks were collected from midvermal anterodorsal lobe VI in 2-month-old mice. PCs are identified by TOM expression (red). vGluT1 (green) and CB1 (blue) expression overlap in PF synapses in LM (***A*–*A*”**). However, in KOs (***C*–*C*”**) CB1 expression is reduced. ***A*’’’**, ***C*’’’**, Zoomed-in views of highlighted regions in ***A*”** and ***B*”**. ***B***, ***D***, Imaris was used to construct surfaces of vGluT1 (green) and CB1 (blue) and to assess volume overlap. ***E***, The ratio of CB1-positive volumes out of the ML ROI is reduced in KOs. ***F***, The volumes of CB1 and vGluT1 overlap are reduced in KOs. ***G***, The ratio of CB1 and vGluT1 overlap over vGluT1 volumes in the ML are reduced in KOs. ML, molecular layer; PC, Purkinje cells; vGluT1, vesicular glutamate transporter 1. Columns show means + SD. An unpaired student *T* test was used to assess *p* values.

In the ML, much of the small punctate “beads on a string” CB1 labeling colocalizes with the granule cells’ presynaptic marker vGluT1 (Fig. 4*A*–*A*’’’), as supported by volumetric analysis of overlapping CB1 and vGluT1 surfaces reconstructed in Imaris from high-resolution confocal stacks (Fig. 4*B*). CB1 ML staining is sparser in Daglα KOs (Fig. 4*C*–*C*’’’), and the ratio of CB1-positive volumes in ML is significantly diminished in KOs (nine LMs, four females and five males; mean, 0.2139; 12 KOs, seven females and five males; mean, 0.1192; a *p* value of unpaired *T* test, 0.0231; the difference between means ± SEM = −0.09464 ± 0.03830; Fig. 4*E*).

The reduction in CB1 ML expression appears to affect predominantly GC presynaptic terminals, while CB1 structures that by morphology are consistent with inhibitory MLI axons appear mostly unchanged in KOs (Fig. 4*C*–*C*’’’). This impression is confirmed by our analysis of CB1 and vGluT1 overlapping voxels that shows a significant reduction in CB1 localization in KOs (nine LMs, four females and five males; mean, 11,848 μm^3^; 12 KOs, seven females and five males; mean, 6,474 μm^3^; a *p* value of unpaired *T* test, 0.0109; the difference between means ± SEM = −5,374 ± 1,905; Fig. 4*F*). Furthermore, the ratio of overlapped CB1 and vGluT1 voxel volumes out of all vGluT1-positive voxels in the regions of interest was also reduced (nine LMs, four females and five males; mean, 0.4158; 12 KOs, seven females and five males; mean, 0.2318; a *p* value of unpaired *T* test, 0.0214; the difference between means ± SEM = −0.1841 ± 0.07341; Fig. 4*G*). The reduced CB1 expression in vGluT1-positive granule cell axons and presynapses may diminish their sensitivity to retrograde eCB signaling.

### The periodic molecular stripe domains in PCs are undisturbed in KOs

**Figure 5.**
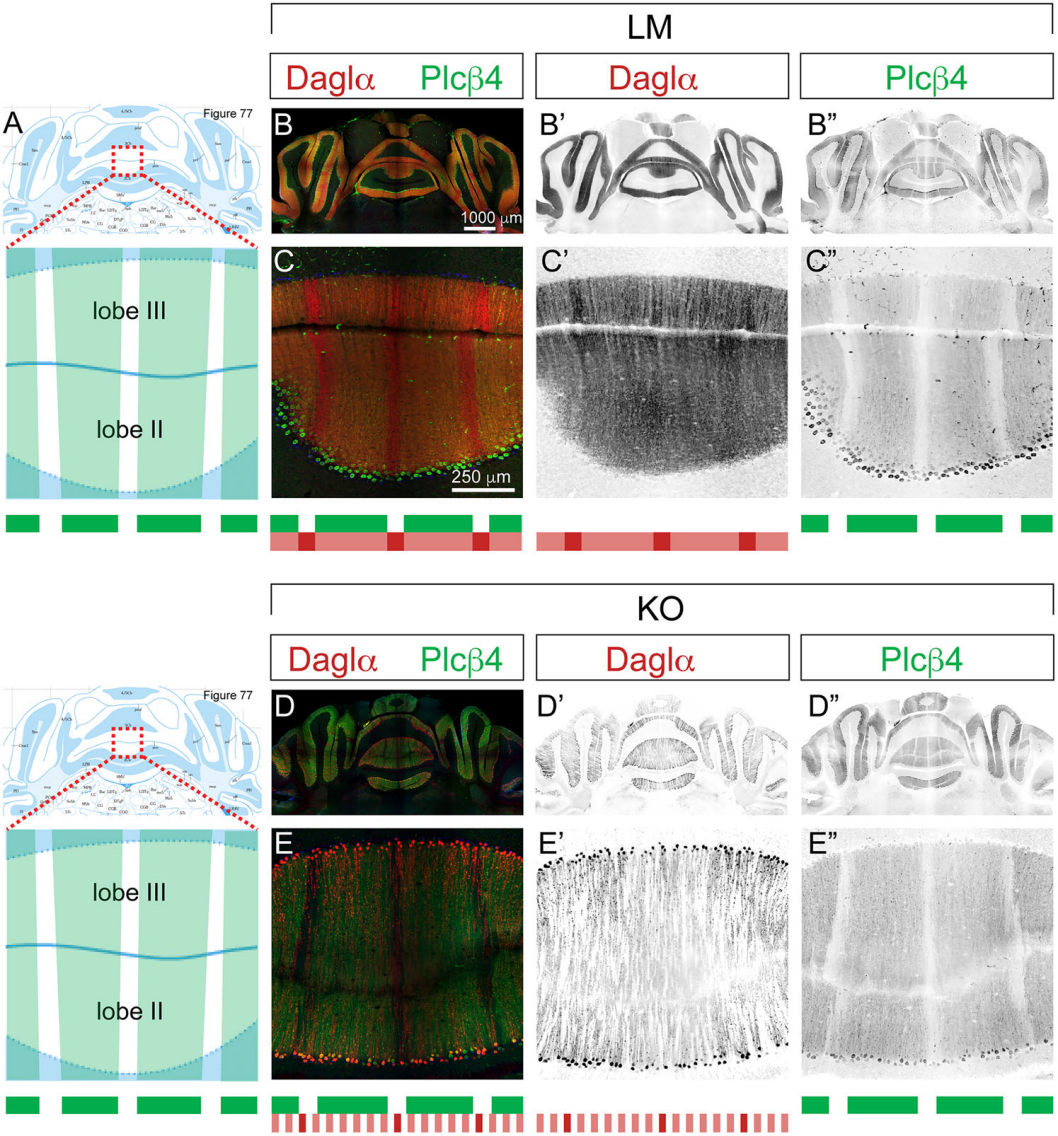
Molecular stripe domains are normal in Daglα KOs. Coronal sections through the anterior zone of cerebellar vermis (lobes II and III) at 2-month-old. ***A***, Figure 77 from Franklin and Paxinos, “The Mouse Brain in Stereotaxic Coordinates,” shows the plane corresponding to the sections analyzed from LMs and KOs, and in the zoomed-in panel, Plcβ4-positive stripe domains are marked with a green mask. ***B***, ***B*’**, Daglα (red) is expressed throughout the PCL in all cerebellar zones and lobes, and (***B*”**) Plcβ4 (green) expression exhibits the typical alternation of positive and negative stripes in the anterior zone. ***C***, ***C*’**, Higher magnification of lobes II–III showing that Daglα expression exhibits stripe-dependent differences in expression levels: (***C*”**) Plcβ4-negative PCs express higher levels of Daglα (summarized in the stripe diagrams below). ***D***, ***D*’**, ***E***, ***E*’**, Patches of Daglα-negative PCs are evident in all cerebellar zones in KOs. However, (***E*”**) the pattern of Plcβ4 stripes is unaffected. Sections from three LMs and three KOs were used in this analysis. Coronal sections from additional animals and higher-magnification images are shown in Extended Data Figure 5-1.

Molecular stripe domains are well characterized in PCs for many physiologically important proteins, including phospholipase C beta 4 (Plcβ4; Fig. 5*B*,*C*,*B*”,*C*”). Plcβ4 plays a key role in the signaling cascades downstream from activation of AMPA and mGluR postsynaptic glutamatergic receptors, stimulating Daglα synthesis of 2-AG upon PC depolarization (Hashimotodani et al., 2005). Immunohistochemistry reveals Daglα expression throughout the PCL, with distinctly different levels of expression in periodic PC clusters (Fig. 5*C*,*C*’). The high-Daglα-expressing PCs localize to Plcβ4-negative stripes, obeying the domain boundaries of the classical molecular stripes (Voogd and Ruigrok, 2004) as identified by Plcβ4 (Fig. 5*A*–*C*”). KOs exhibit patches of Daglα-negative PCs in all cerebellar zones (Fig. 5*D*,*D*’,*E*,*E*’). However, the pattern of Plcβ4 stripes is unaffected (Fig. 5*D*”,*E*”). These observations suggest that the regulation of Plcβ4 expression in PCs is independent of the levels of eCB signaling controlled by Daglα.

10.1523/ENEURO.0400-24.2025.f5-1 Figure 5-1 **Daglα expression in PCs in coronal cerebellar sections in LMs and KOs.** (A-A”) LM, (B-C”) KOs. Top row – rostral, middle row – caudal sections (reference plates from Paxinos and Franklin’s mouse brain atlas are shown in the first column). Bottom row – higher magnification images from lobes VIII-IX. The intensity of Daglα staining varies between neighboring PCs and in different subcellular compartments, *i.e.*, the different dendritic branches and somata. This is particularly noticeable in the Purkinje cells that retain Daglα expression in the KOs. For example, Purkinje cell somata in the KO shown in the middle column exhibit robust Daglα staining, while in the KO shown in the right column, somata are faintly labeled compared to the dendrites. Download Figure 5-1, TIF file.

### Daglα KOs make fewer missteps crossing the horizontal ladder and exhibit normal forelimb coordination

**Figure 6.**
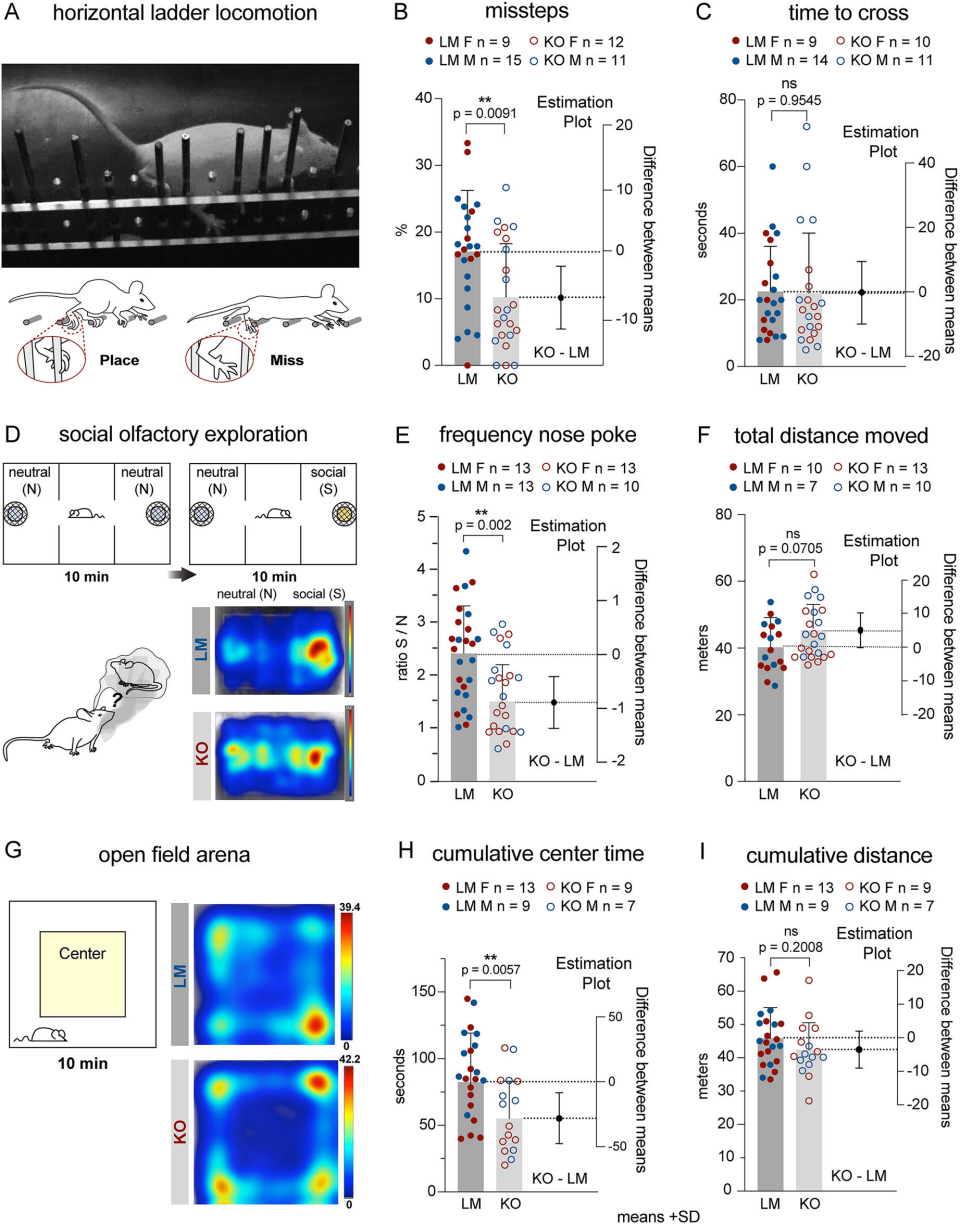
Daglα KOs exhibit increased accuracy of paw placement, decreased social preference, and increased anxiety. ***A***, The precision of paw placement was assessed in mice crossing a horizontal ladder with unevenly spaced rungs. ***B***, KOs had a significantly lower percentage of missteps. ***C***, KOs and LMs took similar times to cross the ladder. ***D***, Social preference was assessed in an olfactory choice test. Representative heatmaps from a LM and a KO show the LM's preference to explore the social cues, while the KO exhibits less preference. ***E***, The preference to explore the social cue (as assessed by the frequency of social over neutral nose pokes) is significantly lower in KOs. ***F***, KOs exhibit increased activity, i.e., longer total distance moved, during the social olfactory exploration test. ***G***, Representative heatmaps show that KOs exhibit increased avoidance of the center in the open-field arena as compared with LMs. ***H***, The cumulative time in the center of the open-field arena is significantly lower in KOs. ***I***, The cumulative distance traveled during 10 min of exploring the open field does not differ between the genotypes. Columns show means + SD. An unpaired student *T* test was used to assess the *p* values.

We evaluated motor coordination and forelimb skilled movement in 2-month-old PC Daglα KO mice. The precision of paw placement was assessed in mice crossing a horizontal ladder with unevenly spaced rungs (Fig. 6*A*). Misses (when the paw slips between or off the rung, resulting in a loss of posture) were scored for hindpaw placement, revealing a significantly lower percentage of misses in KOs (24 LMs, 9 females + 15 males; mean, 16.95%; 23 KOs, 12 females + 11 males; mean, 10.43%; the *p* value of unpaired *T* test, 0.0091; the difference between means ± SEM = −6.520 ± 2.393; Fig. 6*B*). The total time to cross the ladder did not differ between genotypes (23 LMs, 9 females and 14 males; mean, 22.61 s; 21 KOs, 10 females and 11 males mean, 22.33 s, the *p* value of unpaired *T* test, 0.9545; the difference between means ± SEM = −0.2754 ± 4.793; some animals were excluded from total time analysis due to backtracking instead of crossing in one direction; Fig. 6*C*). Fewer missteps on the horizontal ladder suggest increased ongoing adjustments of paw placement in KOs—possibly linking reduced 2-AG signaling in the cerebellum to increased cerebellar output.

**Figure 7.**
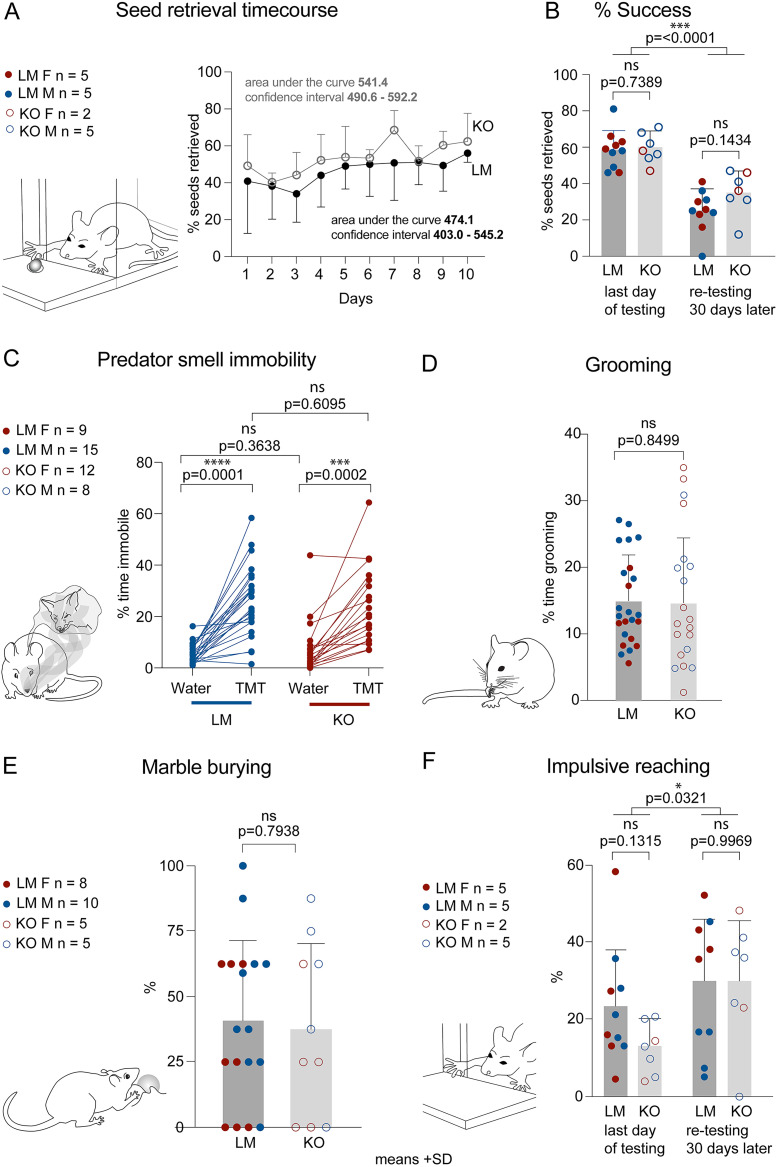
Forelimb skilled movement, predator fear response, and impulsive and repetitive behaviors are unaffected in Daglα KOs. ***A***, The mice were trained to use their forepaws to reach for and retrieve millet seeds through a narrow slit. Their performance was assessed for the percentage of successful reaches over a period of 10 d, revealing no differences between the genotypes in the learning curves. ***B***, There was no difference between the genotypes on the last day of training or in motor memory retention 1 month later. ***C***, KOs exhibit normal fear response as assessed by immobility precipitated by exposure to predator smell. ***D***, Grooming and (***E***) marble burying were used to assess repetitive behaviors, revealing no differences between the genotypes. ***F***, Impulsivity was assessed in the seed-reaching assay by scoring the percentage of impulsive (reaching when there are no seeds on the tray) out of all reaches. The analysis shows no differences between the genotypes. Columns show means + SD. An unpaired Student’s *T* test and ANOVA were used to assess *p* values.

Learning and coordination for the execution of skilled forelimb movements were assessed in the millet seed reaching and retrieval task. In this task, the mice learn a new skill—to reach through a narrow slit to retrieve a millet seed. To evaluate hand dexterity, we assessed the percentage of successfully retrieved seeds for 10 consecutive days after the first 3 d of learning the task (Fig. 7*A*). After 30 d of rest, the mice were reintroduced to the testing chamber to assess their motor memory of the task. We observed no differences between genotypes in the seed retrieval task or in the ability to perform the task 1 month later (Fig. 7*B*).

### Daglα KOs exhibit reduced social preference and increased anxiety but no changes in repetitive and impulsive behaviors or in predator smell-induced fear

**Figure 8.**
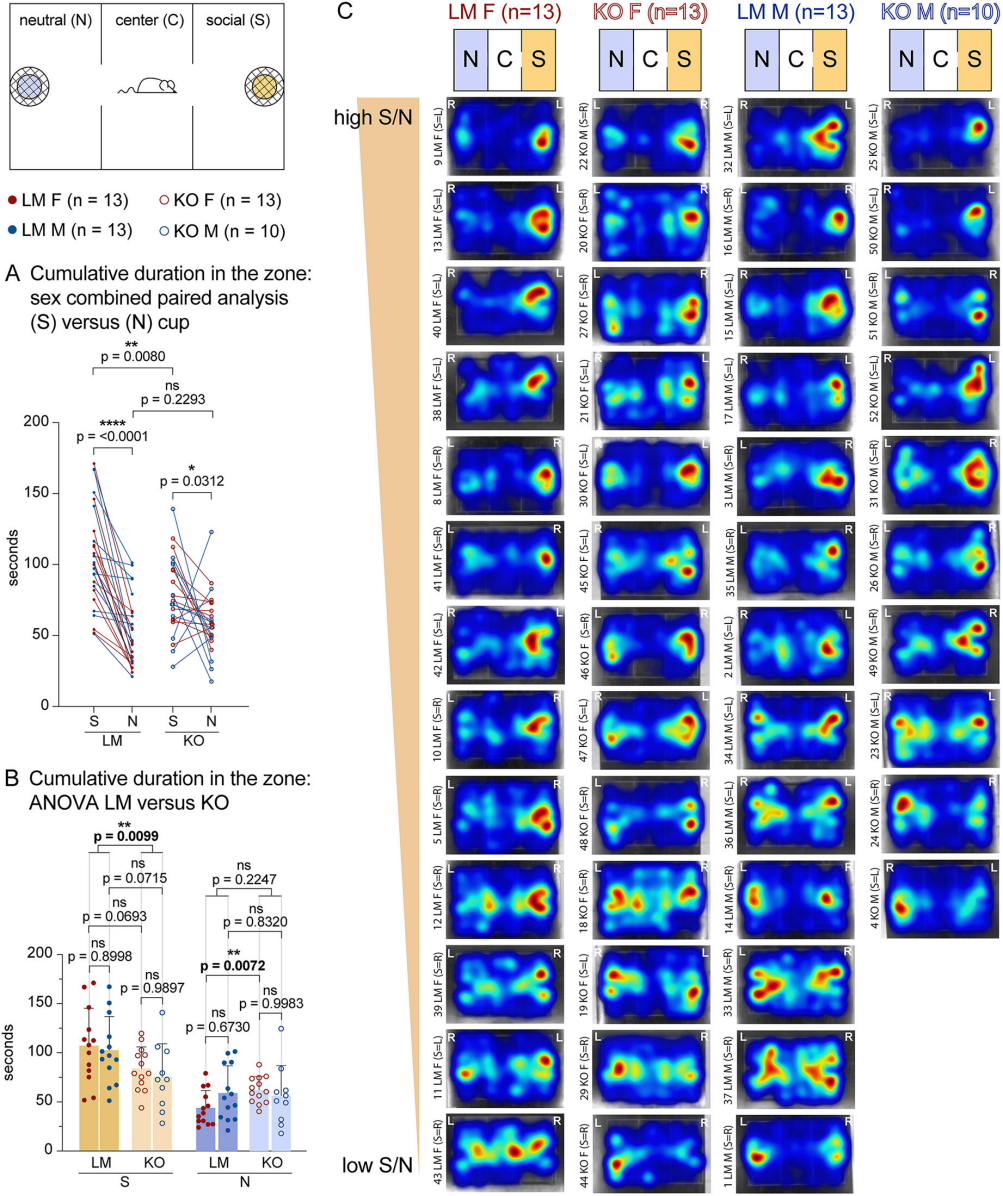
Cumulative duration in social and neutral zones during the social olfactory choice test. Mice were allowed to explore for 10 min a three-chamber arena with a cup containing neutral (clean) cage bedding on the one side and a second cup containing soiled bedding from a cage of unfamiliar mice (social) on the other side, as shown in the diagram. ***A***, Paired analysis of cumulative duration in social (S) versus neutral (N) cup zones. Cumulative durations were assessed automatically in EthoVision XT 17. Most LMs of both sexes spend significantly longer time in the (S) versus (N) cup zone (*p* = <0.0001). In contrast, many KOs of both sexes exhibit a reduced preference for the (S) versus (N) cup zone (*p* = <0.0312). This is primarily due to a reduced preference for KOs to explore the (S) zone (*p* = 0.008 of an unpaired *T* test), while the time to explore (N) zone remains mostly unchanged between the genotypes (*p* = 0.2293 of an unpaired *T* test). ***B***, Detailed statistics, separated by sex and genotype, for cumulative time spent in (S) and (N) cup zones. While the cumulative time that KOs explored the (S) cup is reduced compared with LMs, the cumulative time that KOs explored the (N) cup is comparable or increased (for KO females, *p* = 0.0072), suggesting that KOs are active and willing to explore but show reduced preference to explore social cues. ***C***, These results are shown graphically as heatmaps of cumulative time in different zones of the three-chamber arena. All heatmaps were aligned to show the (S) chamber on the right side, and the locations during testing (right, R; left, L) are shown in white letters. The heatmaps are arranged vertically in the order from highest to lowest S/N preference. Columns show means + SD. Paired *T* test, unpaired *T* test, and ANOVA were used to assess *p* values.

We conducted a social olfactory preference test, where the mouse was placed in a three-chamber arena with two wire cups covering either clean bedding (neutral) or bedding from a cage of unfamiliar age- and sex-matched mice (social). Representative heatmaps illustrate that a LM spends more time exploring the social side of the chamber, while a KO exhibits less preference for the social side (Fig. 6*D*). The preference to explore social cues was assessed by calculating the ratio between the frequency of nose pokes to sniff the social over the neutral cup. LMs exhibit a greater than twofold preference for social over neutral cues, but in KOs, the preference is significantly reduced (26 LMs, 13 females + 13 males; mean, 2.4; 24 KOs, 13 females + 11 males, mean, 1.6; a *p* value of unpaired *T* test, 0.002; the difference between means ± SEM = −0.7570 ± 0.2310; Fig. 6*E*). Decreased preference to explore the social cup in KOs was confirmed by a paired analysis of cumulative durations in social and neutral cup zones (Fig. 8; LMs *p* = <0.0001; KOs *p* = <0.0312). Heatmaps for each mouse showing cumulative duration spent in each location in the three-chamber arena are shown in Figure 8.

KOs exhibited comparable or slightly elevated overall locomotion in the three-chamber arena (Fig. 6*F*), suggesting that the reduction in the percentage of social nose pokes is not due to decreased overall activity. Detailed statistics for cumulative durations in social and neutral cup zones confirm that while the KOs exhibit reduced cumulative time spent exploring the social cup, they spend equivalent or even increased time exploring the neutral cup (Fig. 8).

We conducted an open-field test, which indicated increased anxiety in KOs, who spend less time in the center of the arena than LMs (22 LMs, 13 females + 9 males; mean, 88.18 s; 16 KOs, 9 females + 7 males; mean, 59.21 s; a *p* value of unpaired *T* test, 0.0057; the difference between means ± SEM = −28.97 ± 9.844; Fig. 6*G*,*H*). We did not see a statistically significant difference between genotypes in the total distance moved in the open-field arena (22 LMs, 13 females + 9 males; mean, 46.09 m; 16 KOs, 9 females + 7 males; mean, 42.54 m; a *p* value of unpaired *T* test,  0.2008; the difference between means ± SEM = −3.545 ± 2.722; Fig. 6*I*). To conclude, KOs exhibit reduced social preference and increased anxiety.

Increased anxiety is often associated with altered fear responses. To evaluate predator smell fear-induced immobility in KOs, we compared freezing in mice exposed to the smell of water and TMT, an aromatic compound in fox urine. Our analysis revealed no differences in fear responses between genotypes, suggesting that both predator-induced fear and the sense of smell are normal in KOs (Fig. 7*C*).

We assessed grooming and marble burying to evaluate repetitive behaviors. The percentage of time spent grooming out of 20 min of video-recorded spontaneous behavior did not differ between the genotypes (Fig. 7*D*). Similarly, LMs and KOs did not differ in the percentage of marbles buried (Fig. 7*E*). In addition, we evaluated impulsive behaviors by assessing the ratio of reaches in the seed-reaching task when there was no seed on the tray (i.e., impulsive reaches) and again saw no differences between genotypes (Fig. 7*F*). Our analysis suggests that cerebellar PC-specific Daglα KOs do not exhibit deficits in repetitive or impulsive behaviors.

We conclude that KOs exhibit reduced social preference and increased anxiety but no deficits in predator smell-induced fear nor in impulsive and repetitive behaviors.

### Localization, distribution, and basal synaptic strength of synapses terminating onto PCs are normal in the KOs

**Figure 9.**
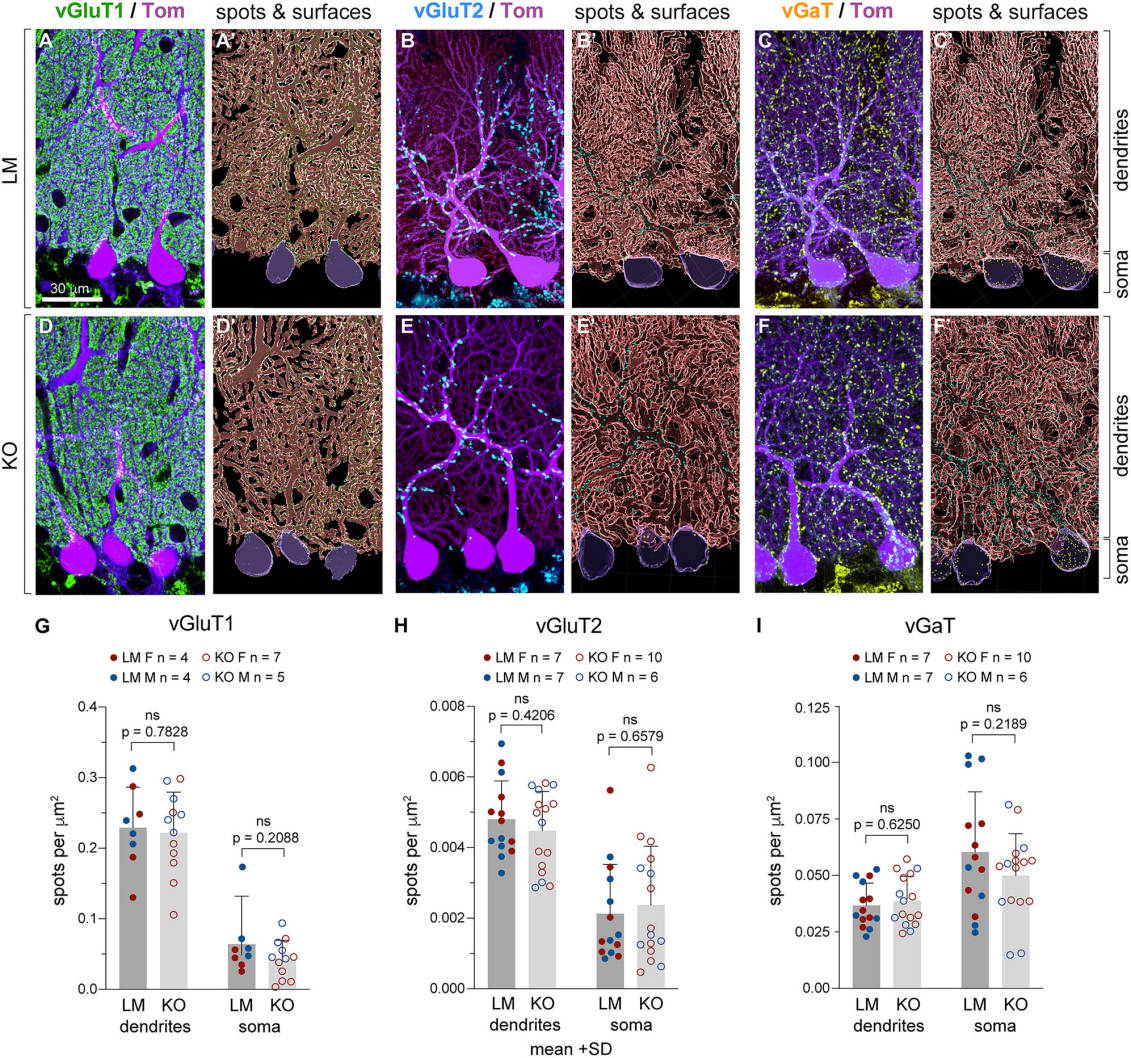
Daglα KO PCs exhibit normal synaptic localization and density. ***A*–*F*’**, Midsagittal cerebellar vermis anterodorsal lobe VI in 2-month-old mice. PCs are identified by TOM expression (purple), GC synapses by vGluT1 (green), CF synapses by vGluT2 (blue), and MLI synapses by vGaT (yellow). Imaris was used to reconstruct PC surfaces and the spots of synaptic puncta, filtered to show only those within 0.2 µm of the PC surface and classified as “on soma” or “on dendrites.” ***G*–*I***, Graphs show the density of synaptic spots per area of dendrites and soma. No significant differences in synaptic density or distribution (soma vs dendrite) were found in Daglα KO PCs. Columns show means + SD. An unpaired Student’s *T* test was used to assess *p* values.

Synaptic competition, retrograde signaling, and neuronal activity regulate the numbers and targeting of excitatory and inhibitory synapses along the developing PC dendrites and somata (Hashimoto et al., 2009; Kalinovsky et al., 2011; Nakayama et al., 2012; Uesaka et al., 2014; Choo et al., 2017). To elucidate whether PC-derived 2-AG plays a role in synaptic placement and the refinement of synaptic territories, we analyzed the density and distribution of excitatory and inhibitory synapses on PC dendrites and somata at 2 months old in the anterodorsal domain of midvermal lobe VI (Fig. 9). PC afferents can be distinguished by their specific expression of presynaptic markers: granule cell axons express vGluT1 and make synapses on PC dendritic spines; climbing fiber synapses localize to PC dendritic shafts and express vGluT2; ML interneurons express vGaT and make synapses on PC somata and dendritic shafts. We collected 5-μm-thick confocal stacks and used Imaris to generate spots from vGluT1, vGluT2, and vGaT synaptic puncta. To quantify the distribution and density of synaptic puncta on PC dendritic and somatic compartments, we filtered the spots to include only those adjacent to the PC surfaces. PC surfaces were generated based on TOM expression in PCs and were separated into dendritic and somatic regions of interest. We found no differences between genotypes in the density or distribution of vGluT1, vGluT2, and vGaT synapses in PC somata and dendrites (Fig. 9), suggesting that the targeting of PC afferent synapses and the overall structural aspects of cerebellar circuitry are normal in Daglα KO PCs.

**Figure 10.**
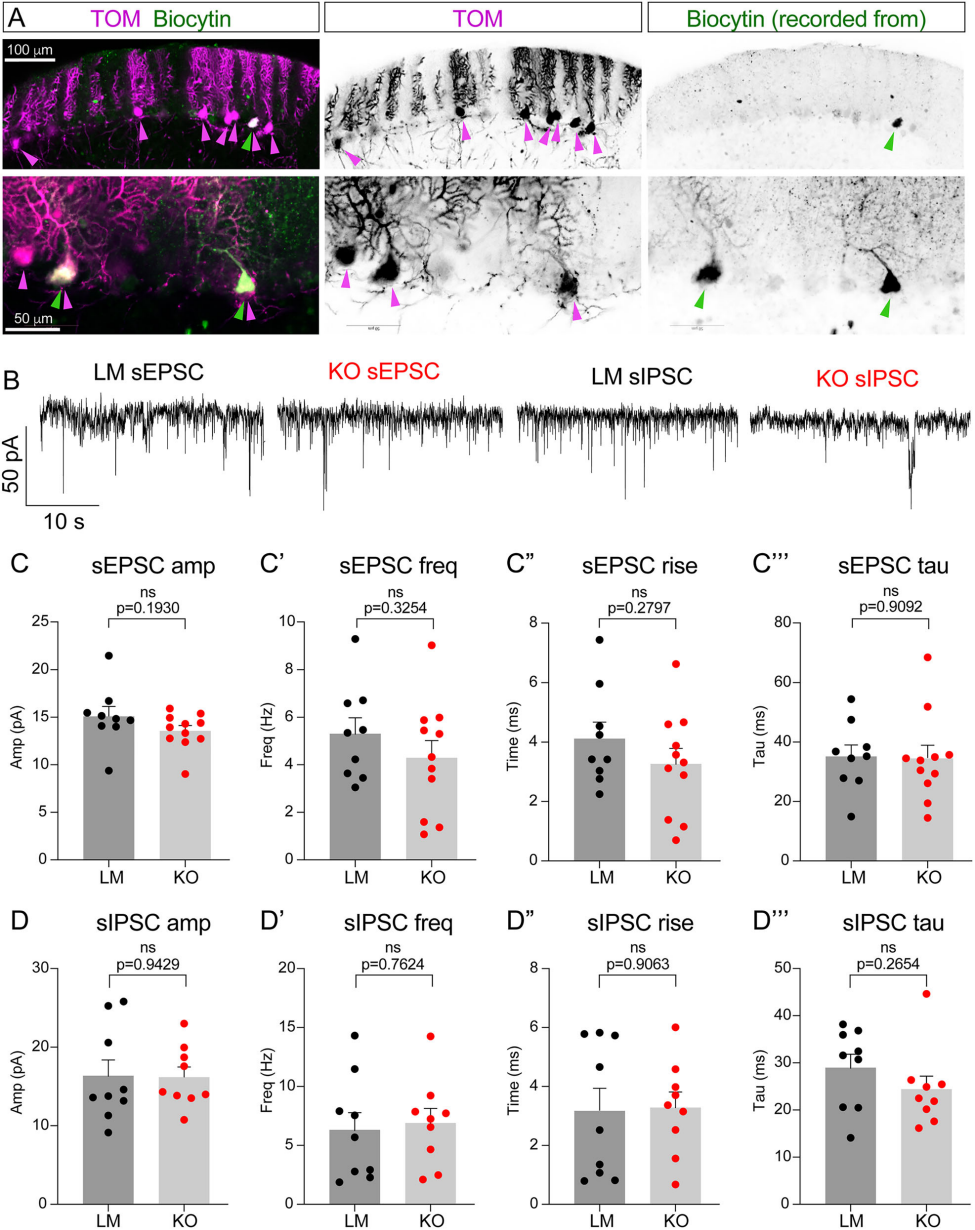
Spontaneous synaptic activity is normal in DAGLα-null PCs. ***A***, Representative images of PCs showing TOM (purple) and biocytin (green) colocalization in recombined PCs from which the recordings were collected. ***B***, Sample traces of sEPSCs and sIPSCs from LM PCs and KO PCs. Daglα KO PCs exhibit no differences in sEPSCs parameters such as (***C***) amplitude, (***C*’**) frequency, (***C*”**) rise time, or (***C*’’’**) tau. ***D*–*D*’’’**, sIPSCs are similarly unaffected. Columns show means + SEM. An unpaired Student’s *T* test was used to assess SEM and *p* values.

To assess whether synapses terminating onto Daglα KO PCs exhibit any functional alterations in basal synaptic strength, we evaluated spontaneous excitatory and inhibitory postsynaptic potentials (sEPSC and sIPSC) in Daglα KO PCs. The recordings were done from cerebellar slices in control TOM-positive PCs in LMs and in TOM-positive Daglα KO PCs in KOs (Fig. 10*A*). Daglα KO PCs exhibit normal basal synaptic strength in both excitatory and inhibitory synapses as assessed by amplitude, frequency, rise time, and decay time (tau) of sEPSCs (Fig. 10*B*,*C*–*C*’’’) and sIPSCs (Fig. 10*B*,*D*–*D*’’’).

In sum, both excitatory and inhibitory synapses on Daglα KO PCs exhibit normal localization and basal functional properties.

### Depolarization-induced short–term synaptic plasticity is dramatically diminished in Daglα KO PCs

Concurrence of synaptic inputs onto PCs controls PC activity patterns and cerebellar-influenced behaviors (Ito, 1972). Retrograde eCB signaling from PCs regulates the strength of all these synapses: PC depolarization triggers an elevation in cytoplasmic calcium, leading to the release of 2-AG that retrogradely activates presynaptic CB1, causing reduced neurotransmitter release from both excitatory synapses (i.e., DSE; Kreitzer and Regehr, 2001b) and inhibitory synapses (DSI; Kreitzer and Regehr, 2001a). Both DSE and DSI recover to baseline after ∼1 min. This timescale is consistent with on-the-fly adjustments of ongoing behaviors likely to regulate the accuracy of paw placement or the persistence in the exploration of social cues. Using global Daglα KOs, DSE and DSI were shown to be dependent on Daglα expression (Tanimura et al., 2010). However, the requirement for PC-specific Daglα expression in cerebellar DSE and DSI has not been investigated before.

**Figure 11.**
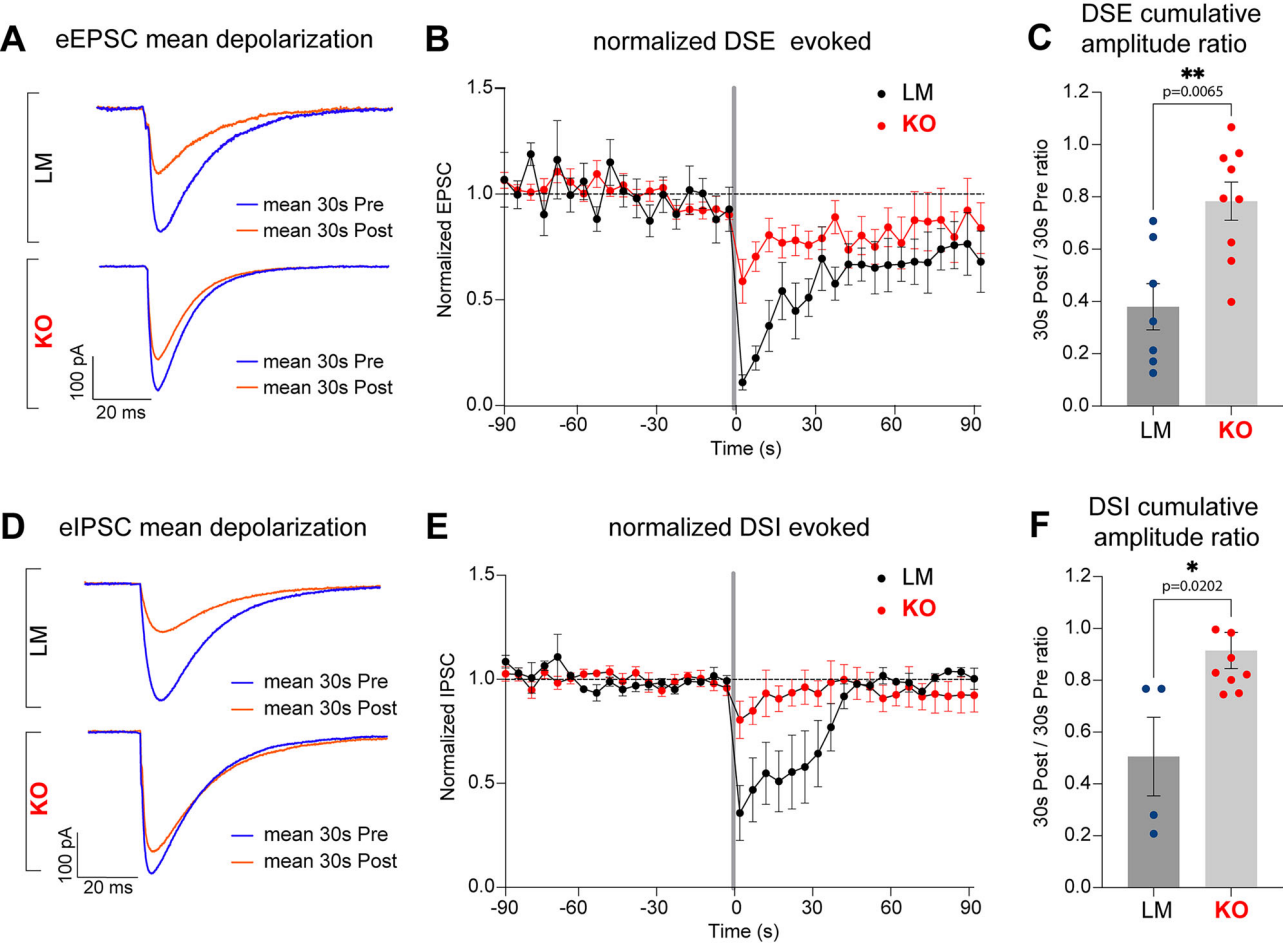
Short-term synaptic plasticity, DSE and DSI, is diminished in Daglα KO PCs. ***A***, Representative eEPSC before (blue, pre) and after (red, post) PC depolarization. LM exhibits smaller post- than pre-eEPSC amplitudes, a hallmark of DSE. However, KOs exhibit almost no difference in pre- and pos-eEPSC amplitudes, i.e., DSE is strongly reduced in the KO. ***B***, Timecourse of the changes in eEPSC amplitudes before and after PC depolarization. In LMs, eEPSC amplitudes are reduced following PC depolarization (Time 0), gradually returning to the baseline over the next 90 s. In the KOs, the reduction in eEPSC amplitudes following PC depolarization is markedly smaller. ***C***, The aggregated cumulative ratio of eEPSCs from 30 s before and 30 s after PC depolarization. LMs exhibit robust DSE, with the postamplitude reduced to less than half of the preamplitude. KOs exhibit small residual DSE, with the post-eEPSCs reduced to ∼0.8 of the pre-eEPSCs. Seven LM and nine KO cells were recorded. ***D***, A much smaller eIPSC amplitude following PC depolarization (red, post compared with blue, pre) indicates DSI in LMs. The difference between pre- and postdepolarization amplitudes in the KO is very small. ***E***, In LMs, timecourse shows robust suppression of eIPSC amplitudes following PC depolarization in LMs, with a return to the baseline within 60 s. In contrast, KOs exhibit very little DSI, with only a small difference between pre- and post-eIPSC amplitudes and a faster return to the baseline. ***F***, The ratio of cumulative eIPSC amplitudes 30 s before and 30 s after PC depolarization, showing that in LMs the post-eIPSCs are about half of the pre, but in KOs there is almost no reduction consistent with dramatically reduced DSI. Four LM and nine KO cells were recorded. Columns show means (±SEM). An unpaired Student’s *T* test was used to assess *p* values.

We assessed DSE and DSI in cerebellar slices by measuring evoked excitatory and inhibitory PSCs (eEPSCs and eIPSCs) in PCs after stimulating PFs and comparing eEPSCs and eIPSCs amplitudes before and after PC depolarization. LMs exhibited more than twofold reduction in eEPSC and eIPSC amplitudes directly following PC depolarization, exhibiting robust DSE and DSI. In contrast, in KOs, eEPSC and eIPSC postdepolarization amplitudes were only slightly diminished compared with predepolarization (Fig. 11*A*,*D*) and returned to the baseline faster (Fig. 11*B*,*E*). In LMs, the ratio of the cumulative eEPSC and eIPSC amplitudes 30 s before versus 30 s postdepolarization shows ∼60% attenuation (Fig. 11*C*,*F*). In contrast, KOs exhibit only very mild (≤20%) attenuation (Fig. 11*C*,*F*; DSE cumulative amplitude ratio, seven LMs; mean, 0.4149; nine KOs; mean, 0.7478; *p* value of unpaired *T* test,  0.0065; the difference between means ± SEM = 0.3330 ± 0.1042; DSI cumulative amplitude ratio, four LMs; mean, 0.5226; nine KOs mean, 0.8996; *p* value of unpaired *T* test,  0.0202; the difference between means ± SEM = 0.3770 ± 0.1389).

**Figure 12.**
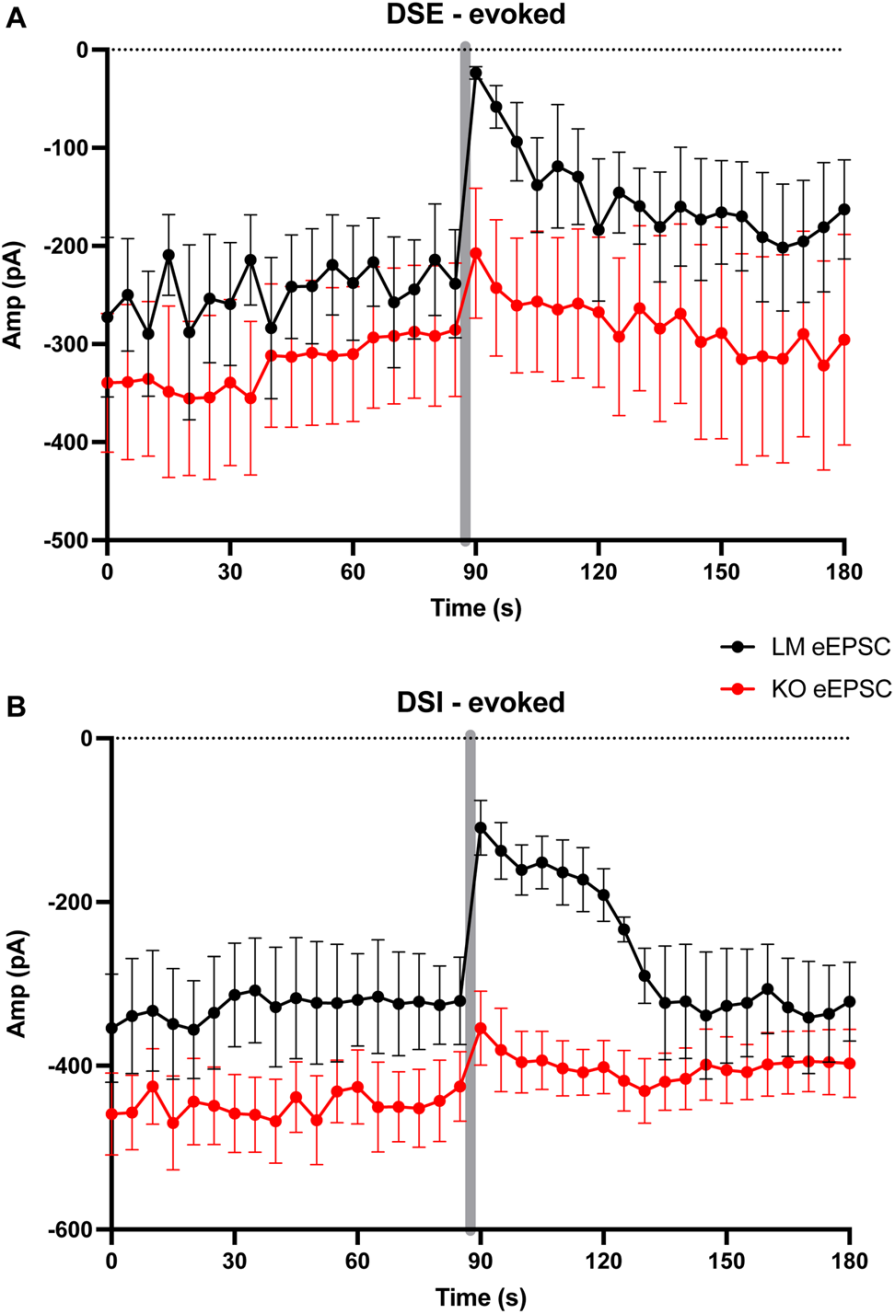
Evoked postsynaptic potentials before and after PC stimulation—not normalized. ***A***, eEPSC before and after PC stimulation in LMs and KOs. ***B***, eIPSC before and after PC stimulation in LMs and KOs.

Figure 12 shows graphs of the same results with non-normalized eEPSCs and eIPSCs. DSE and DSI, which are initiated by robust depolarization, are categorized as eCB-dependent phasic neuromodulation. A distinct form of eCB-dependent synaptic inhibition, which reveals enhanced synaptic strength when eCB signaling is blocked, was shown in cortical and hippocampal inhibitory neurons (Katona and Freund, 2012). Elevated baseline eEPSCs and eIPSCs in Daglα KO PCs, compared with LMs, indicate that PC Daglα may also contribute to the tonic inhibition of excitatory and inhibitory synapses onto PCs.

In conclusion, both DSE and DSI are nearly abolished in Daglα KO PCs, confirming the requirement for PC Daglα expression for these forms of short-term synaptic plasticity.

### KO PCs exhibit reduced cFos expression following social exploration, hinting at the correlation between diminished PC activity and reduced social preference

Due to the recurrent excitatory and inhibitory loops in cerebellar circuits, it is difficult to predict how the combination of greatly reduced DSE and DSI affects PCs’ net activity. We utilized immunodetection of the immediate early gene, c-Fos, as a marker of recent elevated activity in the cerebellar cortex following the olfactory social choice task. c-Fos is a transcription coactivator characterized by rapid turnover and robust upregulation in response to cellular activity, resulting in a sharp peak of maximum expression at ∼1 h after an elevated activity (Morgan and Curran, 1991). We focused on vermal lobes VI–VII, which are involved in social and emotional behaviors (Manzo et al., 2008; García et al., 2015).

**Figure 13.**
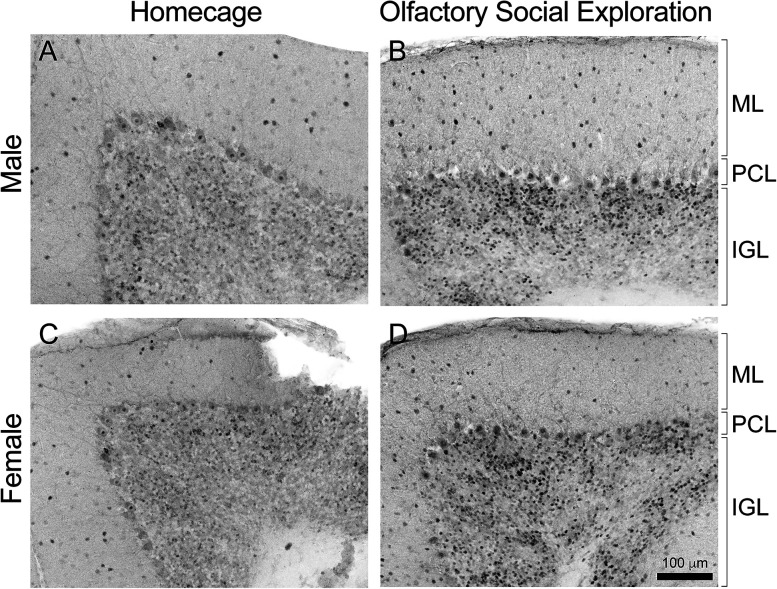
c-Fos expression in lobe VI is elevated following social olfactory exploration. Representative midsagittal sections from lobe VI in wild-type male (***A***, ***B***) and female (***C***, ***D***) mice. In both sexes, increased c-Fos expression is observed in all three layers of lobe VI 1 h after a 15 min exploration of soiled bedding from unfamiliar mice (***A***, ***D***). Cerebellar sections were collected and stained from six male and six female mice (three mice of each sex who remained in their homecage before perfusion and three mice of each sex who explored bedding from unfamiliar mice 1 h before the perfusion).

In wild-type mice, 1 h after a 15 min exploration of soiled bedding from a cage with unfamiliar mice, c-Fos expression in lobe VI is elevated compared with naive mice who remained in their homecages (Fig. 13). In both sexes, increased c-Fos expression is observed in all three layers: the inner granule cell layer (IGL), PCL (contains the monolayer of PC somata), and the ML that contains inhibitory basket and stellate cells. Cerebellar sections were collected and stained from six male and six female mice (three mice of each sex who remained in their homecage before perfusion and three mice of each sex who explored bedding from unfamiliar mice 1 h before the perfusion).

**Figure 14.**
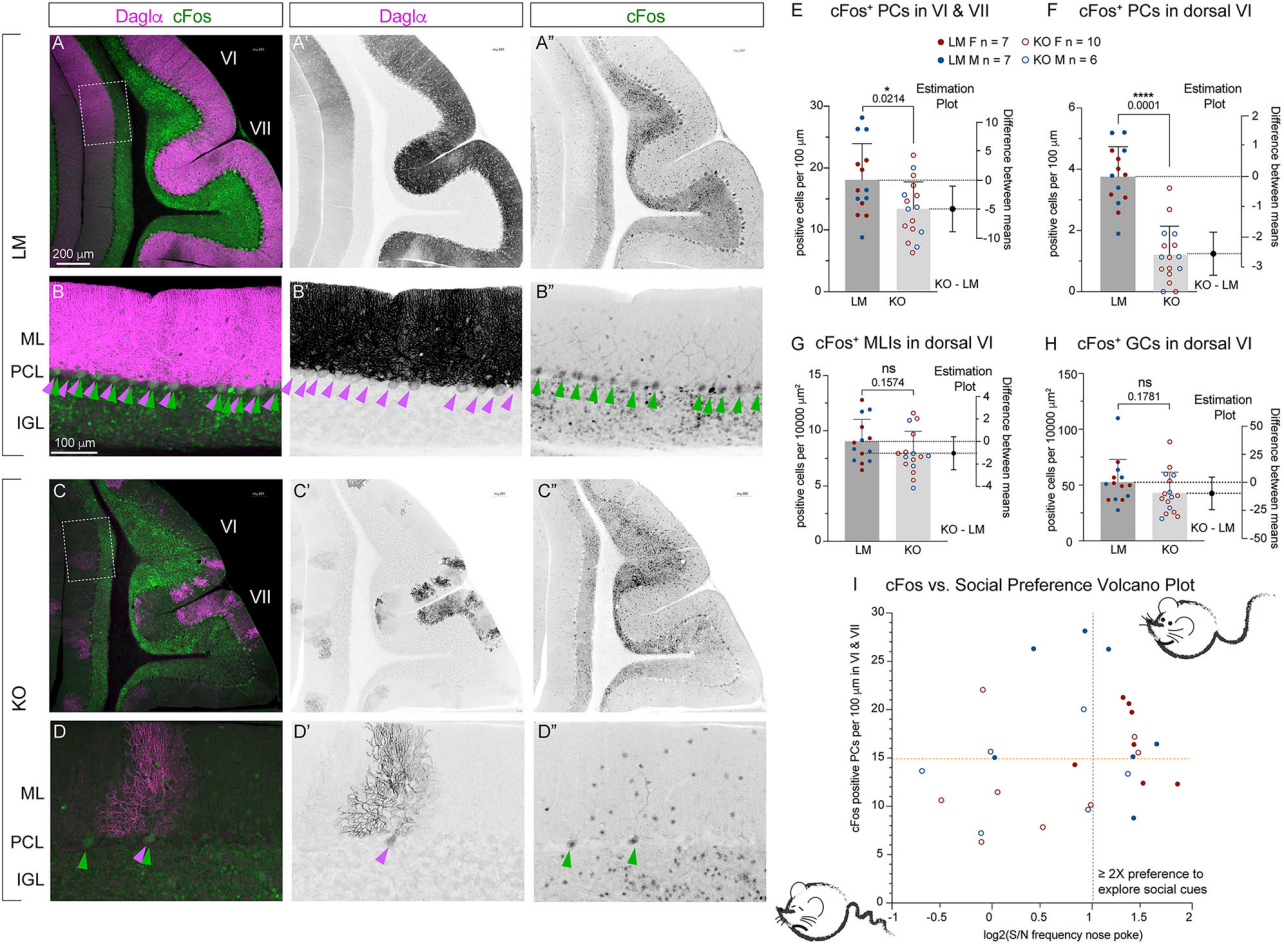
c-Fos expression is high in PCs in midvermal lobes VI–VII after the social olfactory exploration task in LMs but low in KOs. ***A*–*A*”** and ***C*–*C*”**, lobes VI–VII imaged from sagittal midvermal sections. ***A*’**, ***B*’**, Daglα (purple) is highly expressed in most PCs in LMs but (***C*’**, ***D*’**) in only a subset in KOs. ***A*–*B*”**, In LMs following social exploration, c-Fos expression (green) is high in all three layers: granule cells (GCs) in the IGL, PCs (PCs) in the PCL, and ML interneurons (MLIs) in the ML. ***B*–*B*”**, ***D*–*D*”**, Higher-magnification images from anterodorsal lobe VI (dotted rectangles in ***A*** and ***C***). Daglα^+^ PCs are marked by purple arrowheads, and cFos^+^ PC nuclei are highlighted by green arrowheads. ***C*–*D*”**, In KOs, fewer cFos^+^ PC nuclei are evident in the PCL, as evidenced by (***E***) the quantification of cFos^+^ PCs per length of the PCL in lobes VI–VII. ***F***, Higher magnification of the anterodorsal portion of lobe VI (highlighted by dotted squares in ***A*** and ***C***) shows a reduction in cFos^+^ PCs in the Daglα KOs but (***G*** and ***H***) no statistically significant differences in c-Fos expression in GCs and MLIs between genotypes. ***I***, A volcano plot showing a correlation between the density of cFos^+^ PCs in lobes VI–VII and the preference to explore social olfactory cues. LMs exhibit both a greater preference to explore social cues and a higher density of cFos^+^ PCs, with the majority of LM data points clustered in the top-right quadrant of the graph. Columns show means + SD. An unpaired Student’s *T* test was used to assess *p* values.

LMs, similar to WTs, show abundant cFos^+^ cells 1 h after the olfactory social choice test in midvermal lobes VI–VII (Fig. 14*A*,*A*”). As expected, PC Daglα expression is robust in LMs (Fig. 14*A*’). Zoomed-in views of anterodorsal lobe VI (Fig. 14*B*,*B*”; Fig. 14*A*, white rectangle) show that cFos^+^ cells are present in all three layers of the cerebellar cortex (purple arrowheads highlight Daglα expression PCs and green arrowheads point to c-Fos expression in PC nuclei). KOs, as expected, exhibit patches of Daglα-negative PCs (Fig. 14*C*’,*D*’). In KOs, c-Fos expression in IGL and ML is comparable to LMs but significantly lower in PCs (Fig. 14*C*–*D*”). The density of cFos^+^ PCs normalized to the PCL length in lobes VI–VII is lower in KOs as compared with LMs (Fig. 14*E*; 14 LMs, seven females and seven males; mean, 18.08 cells per 100 μm; 16 KOs, 10 females and 6 males; mean, 13.39 cells per 100 μm; a *p* value of unpaired *T* test,  0.0214; the difference between means ± SEM = −4.690 ± 1.924).

The midvermal region of lobes VI–VII likely contains distinct functional zones characterized by specific patterns of c-Fos activation. For consistency between animals, we repeated the analysis focusing on a small anatomical area with well-defined landmarks, the anterodorsal region of lobe VI (Fig. 14*A*,*C*, dotted rectangles). Again, we saw significantly reduced c-Fos expression in KO PCs (Fig. 14*F*; 14 LMs, 7 females and 7 males; mean, 3.755 cells per 100 μm; 16 KOs, 10 females and 6 males; mean, 1.210 cells per 100 μm; a *p* value of unpaired *T* test, < 0.0001; the difference between means ± SEM = −2.544 ± 0.3485). In contrast, quantification of the density of c-Fos expression in the IGL (Fig. 14*G*) and the ML (Fig. 14*H*) demonstrated no differences between the genotypes. A correlation between the density of cFos^+^ PCs in lobes VI–VII and the preference to explore social olfactory cues is supported by the volcano plot showing that the majority of LM data points cluster in the upper-right quadrant of the graph, demonstrating both a greater preference to explore social cues and a higher density of cFos^+^ PCs.

In conclusion, Daglα KO PCs exhibit reduced activity during social olfactory exploration, which correlates with reduced preference to explore social cues and diminished short-term synaptic plasticity.

## Discussion

We generated a PC-specific Daglα KO mouse line to examine the requirement of postnatal PC-derived eCB signaling for the development and function of cerebellar circuits and to evaluate the contribution of the cerebellar eCB signaling to ASD-associated behavioral pathology. This study links deficits in cerebellar eCB signaling to decreased PC activity during social exploration, decreased preference to explore social cues, increased anxiety, and atypical motor coordination. Our results support the key role of PC Daglα in regulating short-term synaptic plasticity and PC activity, highlighting the importance of further investigation of mechanisms that regulate PC activity as potential therapeutic targets for treatments of neurological disorders associated with reduced sociability and increased anxiety. Conversely, despite the robust expression of Daglα in the developing PCs, postnatal ablation of Daglα from PCs does not affect the development of cerebellar circuits, as assessed by the analysis of cerebellar size, layers, and synaptic localization and density.

### PC Daglα KO mouse model highlights the role of cerebellar eCB signaling in the regulation of social behaviors and anxiety

Our results, showing that cerebellar PC-specific Daglα KO decreases social preference and increases anxiety, highlight the role of the cerebellum as an important modulator of social behaviors and emotional states. The eCB signaling system is widely expressed in the brain and plays a key role in the regulation of social and emotional behaviors, as demonstrated by reduced social preference and increased anxiety in global Daglα KO mice (Shonesy et al., 2014; Jenniches et al., 2016). Anatomical regions and cell-type–specific Daglα KO studies highlighted the importance of the hippocampal–amygdalar–limbic axis (Kondev et al., 2023) and dopamine receptor 1 expressing medium spiny interneurons in the striatum (Shonesy et al., 2018) in Daglα-mediated regulation of social preference and anxiety—brain regions that are strongly implicated in social and emotional regulation.

Our work adds to the earlier studies by highlighting the contribution of eCB-regulated cerebellar activity to these behaviors. Our results are not surprising in light of robust functional connectivity between these regions and the cerebellum. Recent work demonstrated direct monosynaptic cerebellar regulation of the reward circuits in the ventral tegmental area (Washburn et al., 2024) and the contribution of these connections to the regulation of social behaviors (Carta et al., 2019; Jackman et al., 2020). In addition, polysynaptic functional connectivity has been described between the cerebellum, the hippocampus, the amygdala (Chao et al., 2023), the medial prefrontal, and the inferior parietal cortex (Buckner et al., 2011; Stoodley and Schmahmann, 2018; Kelly et al., 2020; Van Overwalle et al., 2020; Su et al., 2021), areas of cognitive functions associated with ASD.

### Hypoactivity of PCs in the posterior cerebellar vermis underlies the behavioral phenotypes in PC Daglα KOs

Functional brain imaging in humans identified increased activation of the posterior cerebellar vermis during social and emotional tasks (Van Overwalle et al., 2020), and posterior vermal lesions are associated with social and emotional deficits (Stoodley and Schmahmann, 2018; Chao et al., 2023). In mice, chemogenetic activation or suppression of cerebellar circuits in the posterior vermis alters social interactions (Jackman et al., 2020; Chao et al., 2023). Reinforcing the involvement of this cerebellar zone in the regulation of social behaviors, our study shows reduced c-Fos expression in PCs in lobes VI–VII during social exploration in PC Daglα KOs, highlighting the correlation between PC hypoactivity and reduced preference to explore social cues.

According to the governing hypothesis, the diverse ASD-associated molecular and cellular abnormalities converge on a common pathological mechanism underpinned by the deregulation of the excitatory–inhibitory balance in neural circuits (Rubenstein and Merzenich, 2003; Canitano and Palumbi, 2021). Functional brain imaging in ASD individuals and animal models has predominantly focused on cortical and subcortical regions, highlighting hyperexcitability in the amygdala, frontal, and parietal cortex as ASD hallmarks (Jiang et al., 2022). However, differences in the local configurations of microcircuits can cause the opposite effect downstream of the same molecular changes, suppressing rather than increasing neural excitability. In contrast to increased activity in the cerebral cortex, ASD diagnosis is associated with decreased activity in the cerebellar cortex (reviewed in Wang et al., 2014). Adding to prior research, the results of our study strongly suggest that the predominant contribution of cerebellar dysfunction to reduced sociability stems from the reduced activity of PCs during social exploration.

Reduced activity of PCs enhances cerebellar output (Medina and Khodakhah, 2012), suggesting that the reduced activity of cerebellar PCs can lead to a lower threshold for the cerebellum to generate error messages, causing more frequent adjustments of ongoing behaviors and more switches between behavioral programs—such as increased adjustments of paw placement during horizontal ladder locomotion and increase switching in the exploration between social and neutral cues. This scenario suggests that reduced PC activity in the cerebellar cortex is likely to correspond to increased excitability in the cerebral cortex. Therefore, increasing PC activity in the cerebellum could be a potential future clinical intervention strategy aimed at decreasing cortical hyperexcitability in the context of the relevant neurological disorders.

### Daglα is robustly expressed in the PCs in the postnatal mouse cerebellum

The intensity of Daglα staining is vastly variable between neighboring PCs and subcellular compartments—different dendritic branches and somata. Furthermore, this is particularly noticeable in the PCs that retain Daglα expression in the KOs, possibly because the Daglα labeling is less dense. In some KO animals and cerebellar regions, Daglα staining appears enriched in PC somata compared with LMs (Fig. 5*D*’,*E*’); however, in other KOs it does not. To further clarify this point, we are attaching a figure that includes additional low- and high-magnification images of coronal sections from additional LM and KOs (Extended Data Fig. 5-1). The question of Daglα subcellular localization and its potential redistribution is intriguing. Future studies may investigate whether the variability in Daglα subcellular localization reflects underlying activity, signaling-dependent regulation, or intrinsic molecular differences.

### The development of cerebellar circuitry is unaffected in PC Daglα KOs

A body of research implicates eCB signaling in the regulation of axon growth (Berghuis et al., 2007; Goswami and Hucho, 2007; Keimpema et al., 2010; Jung et al., 2011) and synaptic maturation (Itami et al., 2022; Maroto et al., 2023). Yet, the role of cerebellar Daglα in the structural development of cerebellar circuits has not previously been investigated. The bulk of structural and functional development of cerebellar circuits occurs postnatally. In mice, targeting and refinement of synaptic contacts from granule cells (Lackey et al., 2018), mossy fibers (Kalinovsky et al., 2011), climbing fibers (Kano et al., 2018), and ML interneurons (Ango et al., 2004) progress during the first postnatal month. By ∼P30, around the same time when PC dendritic morphology and firing patterns also acquire mature characteristics (Tano et al., 1992; White et al., 2014), the refinement of synaptic targeting territories of PC afferents and interneurons is also complete, and the molecular stripe domains in PCs assume their mature patterns (Tano et al., 1992; Sillitoe et al., 2009). These aspects of developmental circuit refinement are dependent on PC activity (Lorenzetto et al., 2009; Mikuni et al., 2013; White et al., 2014; Good et al., 2017) and are regulated by retrograde signaling from PCs (Hall et al., 2000; Umemori et al., 2004; Kalinovsky et al., 2011; Uesaka et al., 2014; Choo et al., 2017). The eCB signaling system is expressed in the developing cerebellum (Martinez et al., 2020). Since Daglα-regulated 2-AG production is activity-dependent, it is tempting to hypothesize that PC-derived 2-AG could be an activity-dependent retrograde signal that orchestrates the refinement of cerebellar circuits.

However, in this study, we did not find structural deficits in the postnatal development of cerebellar circuits in PC Daglα KOs: cerebellar midvermal area, localization and density of excitatory and inhibitory synapses onto PCs, and spontaneous synaptic activity are normal in PC Daglα KOs, suggesting that cerebellar circuitry development is intact. Furthermore, PC Daglα KO does not affect PC organization into molecular stripe domains as assessed by Plcβ4 expression, even though the boundaries of stronger versus weaker Daglα expressing PC clusters do obey Plcβ4 stripe domain boundaries. These results suggest that Daglα expression in PCs is dispensable for the postnatal regulation of the development and structural refinement of cerebellar circuits.

The Pcp2 promoter that we used to drive Cre expression turns on just before birth, leaving Daglα expression in PCs intact during embryonic developmental stages, when PCs and their afferents proliferate, differentiate, migrate, and extend axons. Since our experimental design deliberately avoided disruption of eCB signaling at earlier developmental stages or in cell types other than PCs, our conclusions do not exclude the possibility that Daglα may be required for the structural development of cerebellar circuits at earlier developmental stages or in other cell types.

### What cellular mechanisms may contribute to Daglα KOs’ deficits in short-term synaptic plasticity and reduced CB1 expression in vGluT1-positive GC axons?

If local or systemic diffusion of 2-AG contributes significantly to PC short-term synaptic plasticity, it is likely to mask a reduction in 2-AG signaling due to Daglα KO in a subset (Pcp2-positive) of PCs. Nevertheless, Daglα KO PCs exhibit greatly diminished DSE and DSI, confirming that the local activity of Daglα is essential for these forms of short-term synaptic plasticity. The small residual DSE and DSI observed in Daglα KO PCs could be potentially explained by the contribution of other eCB synthesizing enzymes.

In addition to its contribution to short-term synaptic plasticity, prior work has also implicated eCB signaling in the regulation of overall neuronal activity in an autocrine manner (Bacci et al., 2004). Intracellular signaling cascades, energy metabolism, and intrinsic functional properties of Daglα KO PCs could be evaluated in the future to assess the contribution of cell-intrinsic consequences of Daglα KO and the role these changes play in the regulation of neural circuit excitability and behavioral regulation.

CB1 expression levels and localization are reduced following exposure to exogenous agonists or altered levels of eCB signaling (Breivogel et al., 1999; Schlosburg et al., 2010). We assessed the localization of CB1 to vGluT1 puncta in excitatory GC axons and found significantly reduced levels of CB1. Reduced synaptic localization of CB1 is likely to enhance overall excitatory neurotransmission from GCs to PCs—potentially contributing to increased tonic strength and diminished short- and long-term depression in these synapses. We did not quantitatively evaluate CB1 localization to inhibitory synapses in this study, but we expect that it may be less affected since CB1 expression remains prominent along PC dendritic shafts and horizontal trajectories, which is typical for the axons of MLI inhibitory interneurons.

### How do the results of this study expand our understanding of pathology associated with mutations in DAGLα?

The association between rare single gene mutations in *DAGL*α and ASD led to its ranking in the “high-confidence strong candidate autism risk genes category 2” in the gene scoring module in SFARI (https://gene.sfari.org/database/human-gene/DAGLA). Several gene association studies have linked mutations in *DAGL*α (in most cases arising de novo in the patients) to neurodevelopmental disorders (Smith et al., 2017; Bainbridge et al., 2022). In 35 pediatric patients (out of 6,032 probands with neurodevelopmental disorder diagnosis), a significant association was found between rare heterozygote variants in *DAGL*α and ASD, seizure disorders, and ataxia (Smith et al., 2017). A recent study focused on nine idiopathic pediatric patients with mutations in *DAGL*α and a specific set of symptoms: ataxia, nystagmus, and developmental delay (Bainbridge et al., 2022). The two studies together highlight cognitive, social, and motor developmental phenotypes associated with mutations in *DAGL*α. Most of the mutations (point mutations and truncations) described in these studies affect the C-terminal tail of DAGLα that contains phosphorylation sites and Homer-interaction motives, suggesting that subcellular mislocalization or reduction in *DAGL*α activity are sufficient to cause the phenotype. In addition, cerebellar-related symptoms highlighted in these studies strongly suggest the importance of cerebellar eCB signaling in behavioral deficits associated with *DAGL*α mutations.

Our study shows that the reduction of cerebellar Daglα contributes to the symptoms of reduced social preference and anxiety (we did not document seizures or ataxia in our KO mice at the ages examined). These behavioral deficits manifested in concordance with greatly reduced eCB-dependent short–term synaptic plasticity and reduced PC activity but in the absence of obvious anatomical abnormalities in the cerebellum.

## Conclusion

Our results suggest that cerebellar eCB signaling plays a key role in the regulation of cerebellar synaptic plasticity and PC activity, linking deficits in cerebellar eCB signaling to decreased PC activity during social exploration and decreased preference for social cues. These results provide novel evidence for the role of cerebellar eCB signaling in the regulation of social behaviors and hint at therapeutic potential of eCB signaling augmentation or direct stimulation of PC activity for treatments of disorders associated with decreased interest in social interactions. Conversely, despite the robust expression of Daglα in the developing PCs, postnatal ablation of Daglα from PCs does not affect the development of cerebellar circuits, as assessed by the analysis of the cerebellar size, layers, and synaptic localization and density.

## Synthesis

Reviewing Editor: Christie Fowler, University of California Irvine Decisions are customarily a result of the Reviewing Editor and the peer reviewers coming together and discussing their recommendations until a consensus is reached. When revisions are invited, a fact-based synthesis statement explaining their decision and outlining what is needed to prepare a revision will be listed below. The following reviewer(s) agreed to reveal their identity: Valeria Lallai.

This manuscript was reviewed by two experts in the field. Both agreed that the results contribute to the field by showing how loss of Daglα specifically in Purkinje cells affects endocannabinoid signaling. They also acknowledged that it contributes to our understanding for how the endocannabinoid system functions and cerebellar-dependent regulation of behavior.

One outstanding concern was noted by both reviewers. Specifically, in regard to the apparent DAGLa redistribution in PCs that retain DAGLa expression in the KO. It is argued that "We do not see any evidence of redistributed DAGLa localization from the dendrites to the soma in KO PCs or in the neighboring PCs that retain DAGLa expression".

However, in new Fig 5E, E', putative PC somata strongly labelled with DAGLa are evident. These are absent in 5C, C', where DAGLa staining is high, and fairly homogeneous, in PC dendrites, and much less abundant in the somata.

Thus, this needs to be addressed in a revised version of the manuscript. In the consultation session, it was suggested that perhaps the bright somatic signal implies redistribution, so this needs to be fully addressed either in the results section or discussion.

## Author Response

Response to reviewers We greatly appreciate the reviewers' and the editor's supportive evaluations and insightful comments, which have helped us strengthen the manuscript. We revised the manuscript to meet the journal guidelines and addressed all recommendations, as detailed in our point-by-point replies below. Our revisions and comments are highlighted in purple in the responses and in the text of the manuscript.

Synthesis of Reviews:

Computational Neuroscience Model Code Accessibility Comments for Author (Required):

N/A Synthesis Statement for Author (Required):

This manuscript was reviewed by two experts in the field. They both were in agreement that the data presented are interesting and advance the field. However, significant concerns were also noted that need to be addressed in a revision, as outlined below.

Editor Comments:

1. A statistical table with full statistical reporting needs to be included per eNeuro policy. It should include the statistical test performed, statistical value (e.g., F or t value), degrees of freedom, and p value. If two-way ANOVAs are performed, the reporting needs to include both the main effects and the interaction effect. We added a statistics table. In addition to the required statistics reporting, to address questions raised by the reviewer, the statistics table also includes information on breeding generations and which mice were used for multiple experiments.

2. It is noted that some p values were different in figures and text, and number of asterisks is confusing in some cases. Please check all statistics and reporting. We checked all statistics and reporting and updated the text and figures, as suggested by the reviewers. As suggested by the reviewers, we reported the numbers of male and female mice in each group for all graphs and statistical reporting.

3. The methods section should follow after the introduction. The methods section was moved after the introduction.

4. Visual Abstract: The visual abstract is great, but the sparse nature of the genetic manipulation in Purkinje cells should be reflected. We updated the visual abstract as suggested.

Reviewer 1:

The authors studied the role of the eCB synthesizing enzyme diacylglycerol lipase alpha in Purkinje cell (PC) development and function employing a Pcp2-cre / DAGLaf/f mouse line, which results in a mosaic deletion in PCs. They did not find significant anatomical abnormalities in the cerebellum. They did find that DAGLa deletion from a subset of PCs alters motor and social behaviors and reduces Purkinje cell activity during social exploration. Excitatory synapse density onto PCs and spontaneous synaptic transmission were unaltered in Dagla KO PCs. However, eCB-dependant inhibitory and excitatory short-term synaptic plasticity (DSI and DSE) were affected.

This study is comprehensive, combining electrophysiology, behavior, and imaging. Experiments are, in general, well executed, and this study provides important clarification of the cell-autonomous actions of DAGLa in PCs, with potential implications in psychiatric diseases. However, some misleading statements need to be addressed, and additional controls and clarifications are required.

We are grateful for the reviewer's constructive remarks and for acknowledging the quality of our work.

Major:

The use of "PC Daglα KO" is misleading and should be replaced by a term that reflects that DAGLa KO in PCs only affects a fraction of the PCs, as shown in Fig 3 and described by the authors ("Daglα is lost in a subset of PCs in KOs"). This should be clarified throughout the manuscript, including the title, Fig. 3 title, and summary figure. Additionally, I suggest that the characterization in current Fig. 3 is moved to the beginning of the manuscript (after Fig. 1 or, alternatively, combined with it). We clarified in the title that the conditional knockout affects only Pcp2-positive Purkinje cells, checked that this is clear throughout the manuscript, and edited the summary figure to highlight that the recombination affects a Pcp2-positive subset of Purkinje cells. Fig.3 was moved after Fig. 1, and the title was updated as suggested. Consequently, the ordering and numbering of all subsequent figures were also changed.

Total locomotion is not significantly altered in the open field but increased in the social olfactory exploration test in PC-DAGLA KOs mice. A possible explanation is that mice spent less time exploring the neutral or social cues combined. This quantification should be provided. We included a supplemental figure showing the cumulative time spent exploring both social and neutral cups and heatmaps for all the mice in the experiment. Our interpretation of the results (as stated in the manuscript) is that the KOs spend as much or more time exploring the neutral cup as LMs; therefore, overall, the KOs move around the arena as much or more than LMs. The main difference between the genotypes is in the reduced preference to explore the social cup, not in the differences in overall activity. We double-checked the datapoints that were excluded from overall locomotion quantification due to the shadows in the corners of the arena interfering with the automated calculations of total distance traveled (2 experimental days, 9 animals: 3 LM females and 6 LM males). In the updated graph, the trend remains the same, but the p-value is now below statistical significance (p=0.0705 as compared to p=0.0326 in the earlier version). The text has been updated to reflect the absence of statistical significance in the differences between the genotypes for overall locomotion; however, this does not alter our interpretation.

Fig. 3 and Fig. 5: Only representative high-power images of KOs are shown. LMs should be included for comparison. Higher magnification images of LM and KO stained for DAGL and CB1 have been included. The text has been updated in the results section pp. 13-14 and 17 to reflect the changes.

Fig. 6: it would be more appropriate to calculate the proportion of CB1+ VGlut1+ signal rather than the absolute overlapped volume. This is Fig. 4 in the revised version. We provided quantification of the ratio of CB1 out of the ML ROI, as well as of overlapped CB1+vGluT1 volumes out of vGluT1 in the ROIs. We expanded the description of these results.

Fig. 9: It is unclear whether changes in c-fos are due to DAGla-dependent regulation of social behavior. Baseline c-fos levels (in naïve mice) should be assessed. Supplemental Figure 3 and a corresponding paragraph on page 23 of the results have been added to address this omission.

In PCs that retain expression in the KO, DAGLa seems redistributed to the soma (Fig. 4C vs 4E), although this is less obvious in Fig 3 B', C'. This suggests some regulation in DAGLa trafficking in these cells. Fig. 3 - supplement 1 C-C is mentioned but it is unclear what it refers to. This should at least be discussed. We do not see any evidence of redistributed DAGLa localization from the dendrites to the soma in KO PCs or in the neighboring PCs that retain DAGLa expression. In all microscopy that we have done, including Fig. 4 (Fig. 5 in the new version), we see that in the KOs there are DAGLa-negative Purkinje cells, and some Purkinje cells are not recombined, therefore retaining normal expression of DAGLa. Fig. 4 (Fig. 5 in the new version) shows zoomed-out images of coronal cerebellar sections. Since PC dendritic trees are planar, branching extensively in the sagittal plane but capturing <100 micrometer narrow columns in coronal images, each PC dendrite looks like a hairline in the zoomed-out images shown in Fig. 4 (Fig. 5 in the new version). Therefore, with fewer DAGLa-positive PCs, the overall intensity of DAGLa staining in the Molecular Layer is reduced. However, if one looks closely at the Molecular Layer, one sees that in the remaining DAGLa-positive cells, dendrites continue to express high levels of DAGLa.

The authors indicate that "All mice used in this study were maintained on an outbred CD1 genetic background". However, the original mouse lines cited are Bl6. Were all the mice rederived to CD1? Please clarify. All mouse lines arrived in our colony on B6C57 genetic background and have been backcrossed to CD1 background in our colony for {greater than or equal to}7 generations. We added this description to the methods section.

Minor:

The authors pool males and females, since no sex differences are found. This is appropriate but authors should, at least, demonstrate that the sex distribution is not statistically different between genotypes. In response to this suggestion, as well as the comments by the second reviewer, we added information about the numbers of males and females, as well as the specific identity (M vs. F) for each data point, where practical, in all graphs and in the statistics table.

Fig 1A is helpful; however, the description of the breeding strategy lacks reference to the Tdtomato in the results section. Both the methods and the results detail the breeding strategy, including the tdTomato (TOM) reporter mice (pp. 4, 13, 14).

Fig 2E, H: p values are >0.001, therefore (unless notation different from standard, which should be indicated) only 2 asterisks should be used. Thank you for noticing. The number of asterisks (this is now Fig. 6) has been fixed.

Fig. 7: sample traces of sEPSCs and sIPSCs should be shown. We added sample traces to the figure. It is in Figure 8 of the current version.

Fig. 8: P values are different in text and figure. The sample traces are misleading, since DAGLa KO PCs retain substantial (albeit significantly reduced) DSI. Thank you for pointing this out. Statistical reporting has been updated in the text. Thank you for this comment. Sample traces that we showed in Fig. 8 A and D (Fig. 9 A and D in the current version) showed averages over 90 seconds pre-depolarization and 90 seconds post-depolarization. Since the magnitude of DSE and DSI decreases over time faster in KOs than in LMs, the average DSE and DSI appear very small in KOs in these average traces. However, the quantification (that we show in panels C and F) is done for the values averaged over a shorter time (30 seconds pre and 30 seconds post-depolarization). We agree with the reviewer's comment and have replaced the representative traces to be consistent with the quantification in C and F, to show average traces for 30 seconds pre and 30 seconds post.

Supplemental Figure 5 is confusing: what is being plotted? Legend says EPSCs, fig caption text states EPSPs. Please show representative traces. We are sorry for the confusion, but we do not have Supplemental Figure 5. If the question refers to Supplemental Figure 3, we would like to clarify that it shows the same results as Figure 9 plotted without normalizing the amplitudes; therefore, the sample traces are the same as in Figure 9. The text in the figure legend for Supplemental Figure 3 says "EPSC," which is consistent with the data.

Authors argue that "residual DSE and DSI that can be observed in Daglα KO PCs could be potentially explained by the spill-over of 2-AG from neighboring WT PCs". This is unlikely, unless the neighboring WT PC is also depolarized. Thank you for bringing this up; this is a valid point. We changed this in the discussion.

In general, Discussion reads a bit too long and could be more focused. We edited the discussion.

Reviewer 2:

This is a very well-conceived and developed study where the authors generate, describe and characterize a new Purkinje Cell (PC)-specific Daglα conditional KO mouse model for the first time. They use behavioral, histochemical and electrophysiological approaches to demonstrate that selective deletion of Daglα from PC alters motor, social and anxiety-like behaviors and disrupts synaptic plasticity and cell activity, but not the cerebellar anatomy. The methods used are robust, appropriate and well explained.

We are very grateful for the reviewer's positive evaluation of our work and for the insightful and valuable suggestions.

Title:

- Maybe mention something about the synaptic plasticity in the title as it is a key finding of this study We added the wording to the title.

Introduction:

- Page 2: can the authors specify or mention some of the mutations in DAGLα that are associated with neurodevelopmental disorders? We added a short description of the published studies to the indicated paragraph (a new paragraph on top of page 3).

Results:

- My main concern about the results and some of the conclusions is that the authors do not show a measure of the 2-AG tissue levels, be it bulk tissue or locally released (using microdialysis). Thank you for this suggestion. We agree that it would be interesting to assess the levels of 2-AG, other eCBs, and overall lipid metabolism in the cerebella of these KOs. This experiment will be important to generate hypothesis regarding the functional consequences of reduced Daglα expression in PCs, which could guide future studies. However, we think that this experiment is beyond the scope of the current manuscript.

To control for the effectiveness of the genetic manipulation, we show extensive microscopic evidence that Daglα expression is reduced and that the reduction in Daglα expression is specific to the recombined (Cre-positive) PCs. To control for the functional reduction in 2-AG levels, we show greatly reduced DSE and DSI in KO PCs, consistent with published functional evidence that 2-AG signaling is reduced.

- Even though sex differences were not observed, it could be useful to specify the number of male and female mice used in each experiment. Otherwise, one option could be to color-code the scatter plots to distinguish males vs females. We color-coded the data points and added the breakdown into males and females in statistics reporting.

- Figure 1 B-C, please add a magnified image (like in D') to better show the co-localization. We added a figure (Fig. 2 in the new version) to show additional coronal images and higher magnification images for P0 microscopy in order to have a better visual representation of the extent of colocalization.

- In supplemental figure 1 B on the last day of testing for LM the n=10 but on the re-testing day it seems that n is reduced to 9, was one animal excluded? Please report if this is the case. The datapoint for this animal was omitted by mistake (1/5 females). We added this datapoint to the updated graph and updated all statistical reporting. This did not significantly change the results of the statistical analysis (p value in the old graph = p=0.1233, updated p value = 0.1434).

- I would recommend moving panel C of supplement figure 1 at the end (after 1F) so that the figure matches the description of the results in the text. We moved the panels as suggested.

Materials and Methods:

- Please add the total number of animals used in the study. The total number of animals used in experiments is 284, not including animals used for breeding or excluded from analysis.

- It is not always clear whether the same animals went through all the behaviors or if the authors used different animals for different experiments since the reported n. often differs among tests (i.e., Figure 2 B, LM = 24; Figure 2 E, LM=26, Figure 2 H, LM=22). Please clarify. In the statistics table, we have included descriptions of the breeding generations used in each experiment, the instances when the same animals are used in multiple experiments, and reasons for exclusion if any animals are used in one experiment but not in the other.

- Which generation of breeding was used to perform the experiments? We included this information in the statistics table.

- In the immunohistochemistry study, how many animals were used? A total of 107 animals were used in IHC studies: Fig.2: 8 LM P0 mice (2F, 2M sectioned sagittally and 2F, 2M sectioned coronally). The pattern of DAGLa and TOM expression was consistent between the mice, and we did not notice sex differences in expression or recombination. We added this information in the results and figure legend. Fig. 3: 14 LMs (7M and 7F) and 16 KO (10F and 6M). Fig. 4. CB1 and vGluT1 imaging and quantification: 9 LMs (4F and 5M) and 12 KOs (7F and 5M) Fig. 5: 3 LMs and 3 KOs Fig. 7: the same mice as in Fig. 4 S Fig. 4: cFos expression in PCs following olfactory social exploration: 6 male and 6 female CD1-WT mice (3 mice of each sex who remained in their homecages before perfusion, and 3 mice of each sex who explored bedding from unfamiliar mice one hour before the perfusion). Fig. 10: 14 LMs (7F and 7M) and 16 KOs (10F and 6M) - During the grooming, were the animals habituated to the chambers prior to testing? Mice have not been habituated to the chamber prior to the 20-minute video recording session. This clarification has been added to the description of the method.

- In the marble burying test I see that data were collected from 10 KO and 18 LM. Why did the authors use such a different sample size for the 2 groups? These mice are from breeding generation F3. This is all of the LMs and KOs that we had at the time. We did not repeat this experiment with a new cohort of mice since we found no differences between the genotypes in the original cohort.

- Why electrophysiological recordings were performed on mice at PND25-31 while all the other behavioral assessments were performed in 2-month-old mice? PND25-31 is the age that yields the healthiest cerebellar slices for electrophysiology. PCs degenerate quickly in slices from older animals.

Behavioral assessment was performed in young adult animals at the age of two months since we were interested in evaluating social and motor behaviors, which are still immature at PND25-31.

- In Supplemental Figure 1D, since the authors are comparing water vs TMT and the 2 genotypes, I think that a 2-Way ANOVA would be a more appropriate test rather than running 2 separate t-tests. 2-way ANOVA was used. Statistical reporting is clarified in the text and statistics table is attached.

- Figure 8: can the authors clarify how many animals they recorded from? Were multiple cells recorded from the same animal? I am concerned that Figure 8F, n=4 LM might be underpowered, especially because there seems to be a split effect, with 2 cells showing "high" and 2 "low" ratio. We recorded 13 animals overall, 1-4 cells per animal (see the table for the information on the numbers of cells recorded and the type of data collected from each animal). We agree with the reviewer that n=4 for the DSI LMs results exhibit two clusters - two datapoints with high DSI (20% of the original amplitude) and two datapoints with less profound DSI (80% of the original magnitude) - with the average post-amplitude at 40% of pre. This suggests that our analysis is underpowered, underrepresenting the significance of the KO effect. This is a limitation that we would like to address in future studies.

1 LM Mouse 1 3 cells 3 sEPSC 3 DSE LM 2 LM Mouse 2 4 cells 4 sEPSC 2 DSE LM 3 LM Mouse 3 3 cells 2 sEPSC + 1 sIPSC 2 DSE LM 4 LM Mouse 4 5 cells 5 sIPSC 2 DSI LM 5 LM Mouse 5 3 cells 3 sIPSC 2 DSI LM 6 KO Mouse 1 1 cell 1 sEPSC 1 DSE KO 7 KO Mouse 2 4 cells 4 sEPSC 3 DSE KO 8 KO Mouse 3 2 cells 2 sIPSC 2 DSI KO 9 KO Mouse 4 4 cells 4 sIPSC 3 DSI KO 10 KO Mouse 5 3 cells 3 sEPSC 3 DSE KO 11 KO Mouse 6 1 cell 1 sIPSC 1 DSI KO 12 KO Mouse 7 3 cells 2 sEPSC + 1 sIPSC 2 DSE + 1 DSI KO 13 KO Mouse 8 2 cells 1 sEPSC + 1 sIPSC 1 DSE + 1 DSI KO
