# Supplementary figures and images for: Deletion of Endocannabinoid Synthesizing Enzyme DAGLα in Pcp2-Positive Cerebellar Purkinje Cells Decreases Depolarization-Induced Short–Term Synaptic Plasticity, Reduces Social Preference, and Heightens Anxiety
Source: eNeuro. 2025 Jul 22;12(7):ENEURO.0400-24.2025. doi: 10.1523/ENEURO.0400-24.2025 (PMC12308783; doi:10.1523/ENEURO.0400-24.2025)

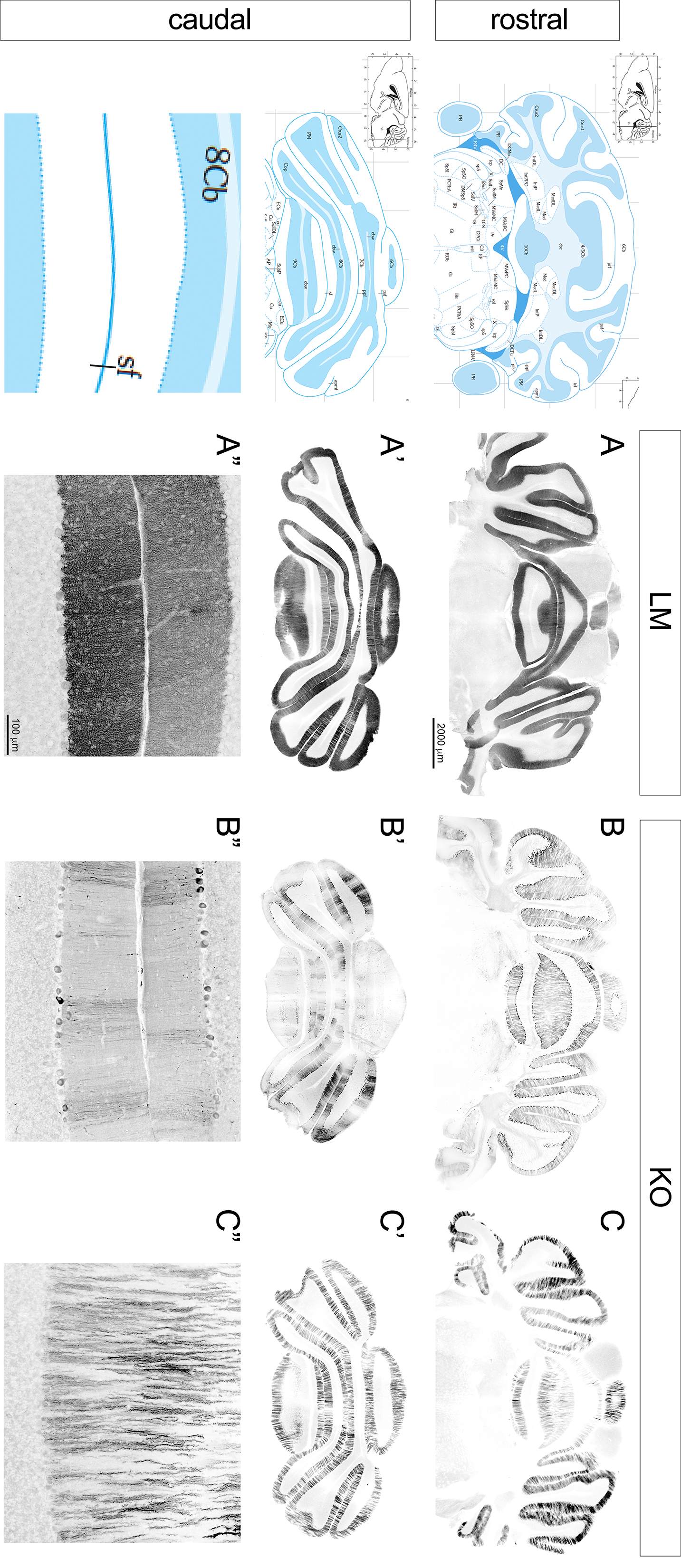

Supplement: Figure 5-1 — Daglα expression in PCs in coronal cerebellar sections in LMs and KOs. (A-A”) LM, (B-C”) KOs. Top row – rostral, middle row – caudal sections (reference plates from Paxinos and Franklin’s mouse brain atlas are shown in the first column). Bottom row – higher magnification images from lobes VIII-IX. The intensity of Daglα staining varies between neighboring PCs and in different subcellular compartments, i.e., the different dendritic branches and somata. This is particularly noticeable in the Purkinje cells that retain Daglα expression in the KOs. For example, Purkinje cell somata in the KO shown in the middle column exhibit robust Daglα staining, while in the KO shown in the right column, somata are faintly labeled compared to the dendrites. Download Figure 5-1, TIF file. [file eneuro-12-ENEURO.0400-24.2025-s002.tif]
